# Mucoadhesive Peptide‐Catalase Self‐Assembled Nano‐Formulation for Effective Treatment of Mucosal Inflammatory Diseases

**DOI:** 10.1002/advs.76390

**Published:** 2026-07-02

**Authors:** Xinzhu Li, Yuxuan Ma, Di He, Yingying Huang, Yutong Jiang, He Zhao, Shengjie Ge, Ruirui Zhao, Hejia Yuan, Wendi Jiang, Wenjun Zhu, Kai Liu, Han Zhang, Zhuang Liu

**Affiliations:** ^1^ Institute of Functional Nano & Soft Materials (FUNSOM) Soochow University Suzhou Jiangsu China; ^2^ College of Pharmaceutical Science Soochow University Suzhou Jiangsu China; ^3^ Wisdom Lake Academy of Pharmacy Xi'an Jiaotong‐Liverpool University Suzhou Jiangsu China; ^4^ Institute of Biomedicine and Biotechnology, Shenzhen Institutes of Advanced Technology Chinese Academy of Sciences Shenzhen Guangdong China

**Keywords:** acute lung injury, interstitial cystitis, inflammation, local administration, mucoadhesion, reactive oxygen species

## Abstract

Mucosa, a vital interface between the body and external environment, often suffers from reactive oxygen burden within its microenvironment, leading to various mucosal inflammatory diseases. Drug delivery directly to inflamed mucosal regions offers a promising therapeutic approach, yet efficacy is compromised by inherent physiological clearance mechanisms. Herein, we developed a covalent mucoadhesive nanoantioxidant self‐assembled by a cysteine‐modified short peptide (CR_8_L_10_) and catalase (CAT) for the treatment of mucosal inflammatory diseases. The obtained CR_8_L_10_@CAT nanocomplexes with a cysteine‐decorated surface enable robust mucoadhesion by forming dynamic disulfide bonds with mucin‐rich mucosa, leading to significantly enhanced CAT retention. Upon intravesical instillation, CR_8_L_10_@CAT with improved urine‐resistant bladder retention, could be used to treat hard‐to‐manage interstitial cystitis/bladder pain syndrome (IC/BPS). Notably, intravesically administered CR_8_L_10_@CAT effectively eliminated excessive reactive oxygen species (ROS) in the bladder mucosa, thereby inhibiting pro‐inflammatory responses, restoring urothelial integrity, and alleviating pain and voiding dysfunction, demonstrating significantly better analgesic effects and superior functional improvement than clinically used intravesical agents. Additionally, inhalation of the mucoadhesive CR_8_L_10_@CAT nanoantioxidant also showed enhanced pulmonary retention to effectively mitigate ROS‐associated inflammation in treating acute lung injury (ALI). This mucoadhesive CR_8_L_10_@CAT nanoantioxidant represents an effective therapeutic strategy to manage different mucosal inflammatory diseases, holding great promise for clinical translation.

## Graphical abstract

CR_8_L_10_ is self‐assembled with catalase (CAT) to form CR_8_L_10_@CAT nanocomplexes, which can anchor to mucosal surfaces via thiol‐disulfide exchange with mucin, thereby enhancing their mucosal retention. The efficient reactive oxygen species (ROS) scavenging at inflamed sites would then result in successful treatment in both the intravesical IC/BPS model and the inhaled ALI model.

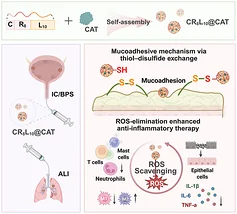

## 1 Introduction

Mucosa is the soft tissue that lines the body's canals and organs in the respiratory, digestive, and reproductive systems. Acting as the first line of defense, the mucosa is often threatened by pathogens, irritants, and metabolic stressors, leading to mucosal inflammatory diseases. Previous studies have demonstrated that the oxidative stress induced by the excessive production of reactive oxygen species (ROS) is an important contributor to mucosal inflammatory diseases [1, 2, 3]. In the inflammatory microenvironment, immune cells and epithelial cells produce excessive ROS, which directly induces intracellular oxidative stress damage to proteins, lipids, and nucleic acids, ultimately leading to cell apoptosis [4]. Furthermore, overproduced ROS can activate multiple pro‐inflammatory signaling pathways, resulting in the upregulation of various pro‐inflammatory cytokines. These factors not only contribute to the disruption of the epithelial barrier but also lead to the recruitment and activation of pro‐inflammatory immune cells such as neutrophils and macrophages. In turn, these immune cells further exacerbate ROS generation, establishing a self‐amplifying feed‐forward loop that perpetuates oxidative stress and inflammatory burden [1, 5, 6].

Specifically, interstitial cystitis/bladder pain syndrome (IC/BPS) is a chronic inflammatory bladder disorder characterized by pelvic pain, urinary urgency, and frequency that profoundly compromises quality of life. Clinical evidence has identified that excessive ROS is a key pathological driver in IC/BPS, contributing to chronic bladder inflammation through multiple mechanisms, such as activation of the NF‐κB/TLR4 and MAPK/JNK signaling pathways, induction of chemokine‐mediated immune cell recruitment, and sensitization of nociceptive pathways [2, 7]. In clinical practice, intravesical instillation of dimethyl sulfoxide (DMSO), hyaluronic acid (HA), or hyaluronic acid/chondroitin sulfate (HA/CS) constitutes the current standard‐of‐care for IC/BPS [8, 9]. Unfortunately, by acting through indirect anti‐inflammation, analgesic, or urothelial repair mechanisms, these therapies fail to target the underlying pathological causes, resulting in only transient and inconsistent benefits. Therefore, sustained elimination of excessive ROS within the mucosa represents a promising approach to be explored for effective treatment of IC/BPS and other mucosal inflammatory diseases.

In recent years, a variety of natural antioxidative enzymes (e.g., catalase and superoxide dismutase) and inorganic nanozymes that mimic antioxidant enzyme activities (e.g., cerium oxide nanoparticles and manganese oxide nanoparticles) have been investigated for the treatment of inflammatory diseases [10, 11, 12]. Among these antioxidative agents, catalase (CAT), an endogenous enzyme, decomposes hydrogen peroxide (H_2_O_2_) into water and oxygen, offering superior catalytic activity (with a catalytic rate of up to 10^7^ molecules per second), excellent biocompatibility, and ready availability [13]. CAT‐based nanotherapy systems, such as CAT‐encapsulated mesenchymal stem cell‐derived nanovesicles [14], CAT‐loaded PLGA nanoparticles [15], and thiolated chitosan‐assembled CAT nano‐formulation [16], have shown promise in eliminating ROS for the treatment of various inflammatory diseases.

Local administration offers distinct advantages for mucosal inflammatory diseases by delivering therapeutics directly to the site of inflammation, thereby maximizing local drug exposure and minimizing systemic toxicity [17, 18]. However, in mucosal inflammatory diseases, the therapeutic efficacy of local administration is often compromised by multiple physiological clearance mechanisms that rapidly eliminate drugs from mucosal surfaces and reduce their retention in mucosal tissues [19, 20]. For instance, intravesical instillation is the standard clinical procedure for treating bladder diseases such as IC/BPS and bladder tumors. Unfortunately, over 90% of the instilled drug is expelled from the bladder within 2 h by urine flushing, and little drug remains after 24 h, significantly limiting the therapeutic efficacy [21, 22]. Therefore, there is an urgent need to develop novel delivery strategies that overcome physiological clearance barriers and enable efficient delivery of therapeutics to the disease site.

Short peptides, as simplified and programmable building blocks, possess well‐defined structures, facile synthesis and functionalization, adaptive drug loading, and favorable biocompatibility and biodegradability, making them highly advantageous delivery platforms with strong translational potential. Herein, we designed a cysteine‐functionalized short peptide (CR_8_L_10_) as a novel platform for the covalent mucoadhesive delivery of CAT to inflamed mucosa across multiple tissues (Scheme 1). It was found that CR_8_L_10_ could readily self‐assemble with CAT into well‐dispersed nanocomplexes (CR_8_L_10_@CAT) with greatly enhanced mucosal retention, by engaging in dynamic thiol‐disulfide exchange with mucin networks on the mucosal surface. Upon intravesical instillation, CR_8_L_10_@CAT demonstrated excellent urine‐resistant bladder retention and ROS‐scavenging capability by continuously decomposing diffusible ROS at the mucosal interface, leading to suppressed inflammatory responses, restored urothelial barrier integrity, as well as improved pain‐related behaviors and voiding functions in mice with IC/BPS. We further elucidated the anti‐inflammatory mechanisms, which involved the regulation of the ROS metabolic pathway, NF‐κB/TLR4 and MAPK/JNK signaling pathways, chemokine‐mediated signaling pathways, and nociception‐related pathways. In comparison with clinically used intravesical instillation agents, including DMSO, HA, and HA/CS, CR_8_L_10_@CAT showed clearly superior therapeutic efficacy. Furthermore, in a mouse model of acute lung injury (ALI), in which ROS also plays a central pathogenic role by inducing cell damage and activating pro‐inflammatory cascades, inhalation of CR_8_L_10_@CAT nanoantioxidant significantly enhanced pulmonary retention and effectively attenuated ROS‐driven inflammation. Therefore, the cysteine‐modified short peptide carrier developed in this work is a versatile platform to enable mucosa‐retained antioxidant enzyme delivery, offering transformative potential for addressing a broad spectrum of mucosal inflammatory disorders.

**SCHEME 1:**
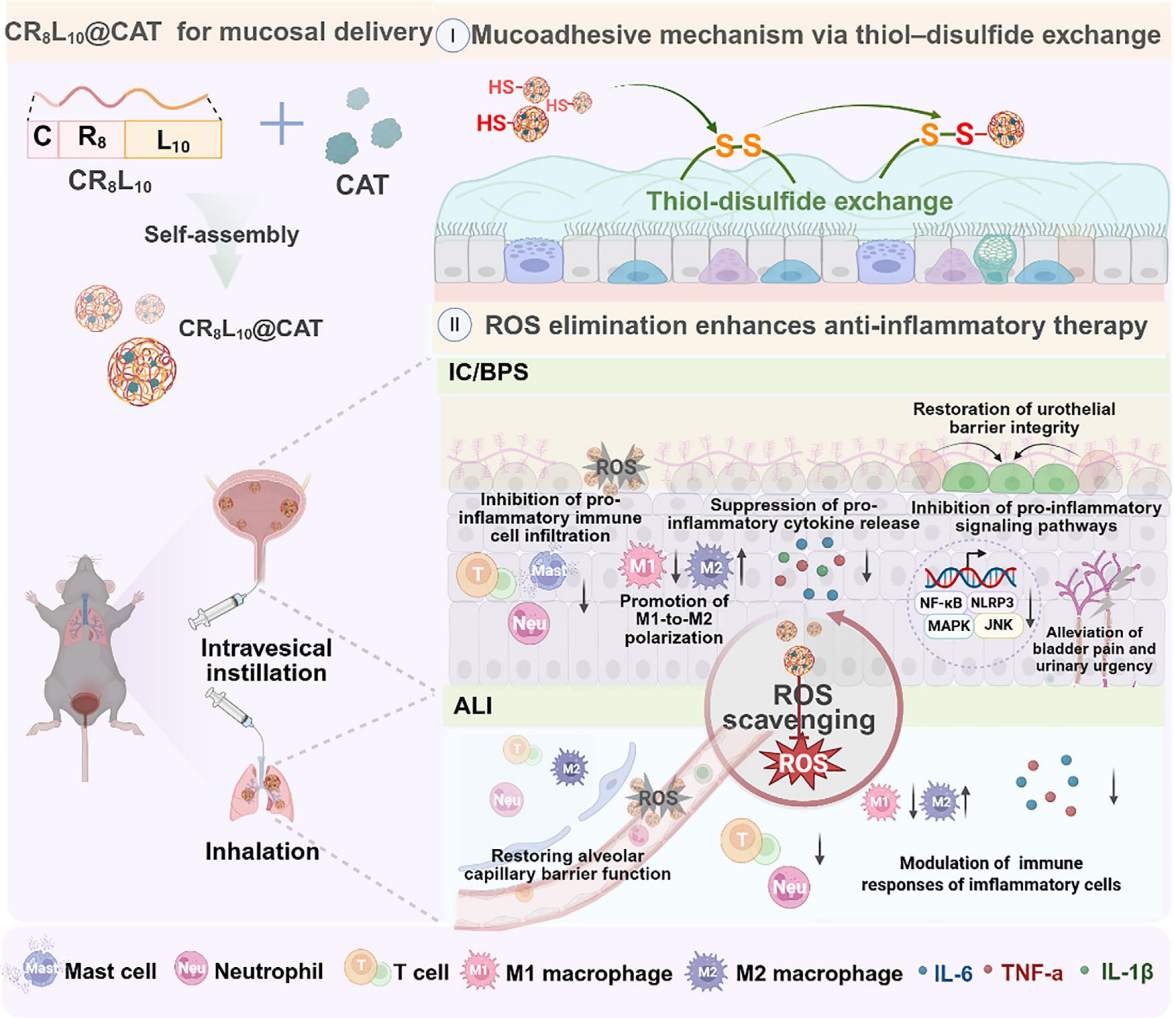
Schematic illustration of CR_8_L_10_@CAT as a mucoadhesive nanoantioxidant for the treatment of mucosal inflammatory diseases such as IC/BPS and ALI. CR_8_L_10_ is self‐assembled with catalase (CAT) to form CR_8_L_10_@CAT nanocomplexes, which can anchor to mucosal surfaces via thiol‐disulfide exchange with mucin, thereby enhancing their mucosal retention. Efficient reactive oxygen species (ROS) scavenging at inflamed mucosal sites subsequently suppresses inflammatory responses and supports therapeutic efficacy in both the intravesical IC/BPS model and the pulmonary ALI model.

## 2 Results and Discussion

### 2.1 Preparation of CR_8_L_10_@CAT Nanocomplexes

Considering conventional mucoadhesive systems are based on the cationic carrier [23, 24], we initially designed an amphiphilic cationic peptide R_8_L_10_. Next, cysteine was conjugated to the N‐terminus of R_8_L_10_ to generate CR_8_L_10_. Subsequently, R_8_L_10_ and CR_8_L_10_ were co‐assembled with CAT at various peptide: CAT weight ratios (15:1, 10:1, 5:1, and 1:1) through cooperative non‐covalent interactions, mainly including electrostatic interactions, hydrophobic effects, and hydrogen bonding, forming R_8_L_10_@CAT and CR_8_L_10_@CAT nanocomplexes, respectively (Figure 1A). For both peptide‐CAT systems, dynamic light scattering (DLS) measurements showed that the hydrodynamic diameter decreased with the rise of the peptide: CAT weight ratio from 1:1 to 10:1, but increased again at 15:1. Among these formulations, the 10:1 peptide: CAT ratio produced nanocomplexes with a relatively small particle size of 177.4 ± 15.4 nm and a low polydispersity index (PDI) of 0.21 (Figure 1B).

**FIGURE 1:**
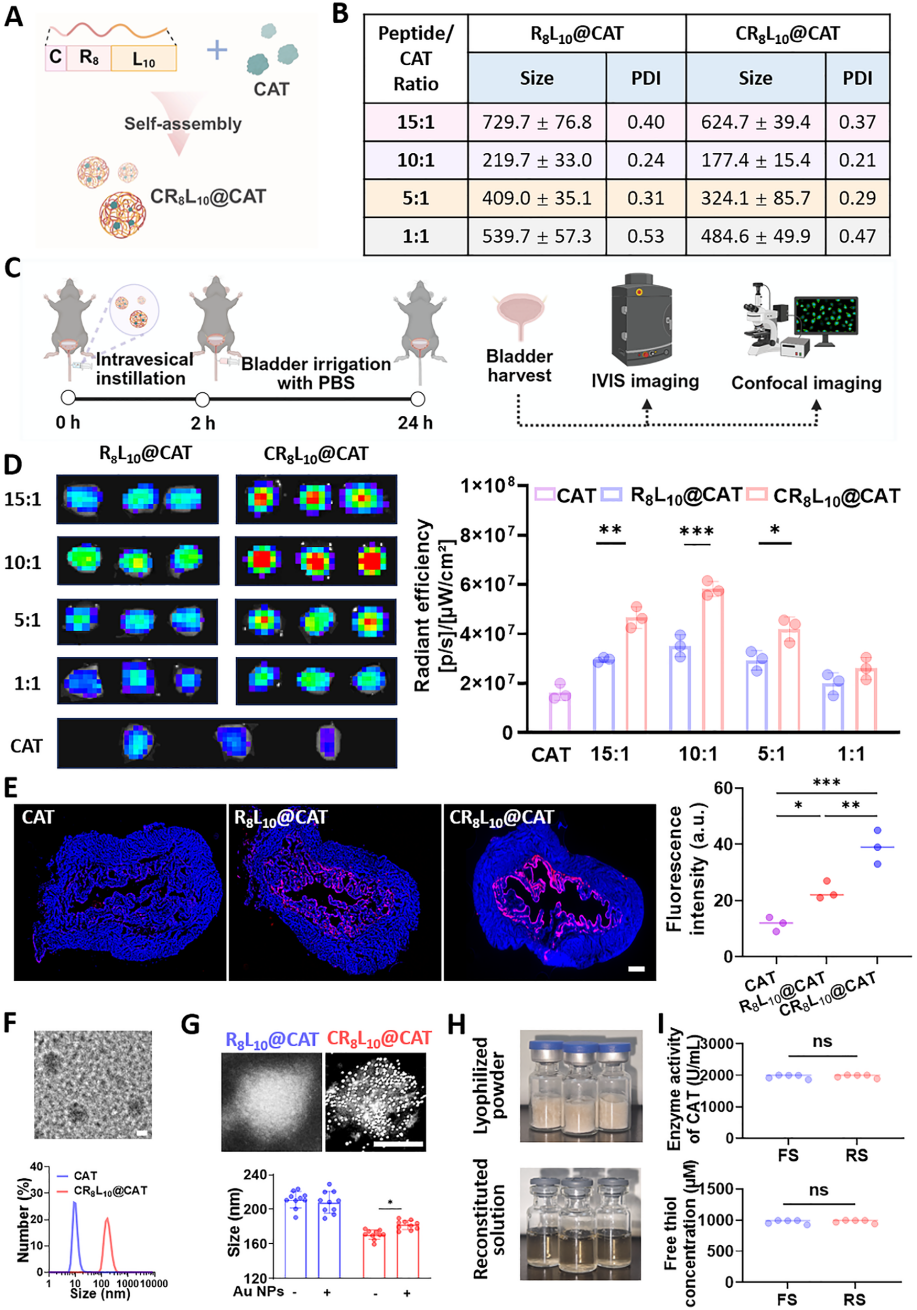
Preparation and screening of CR_8_L_10_@CAT nanocomplexes. (A) Schematic illustration of the self‐assembly of CR_8_L_10_@CAT nanocomplexes from cysteine‐modified peptide CR_8_L_10_ and CAT. (B) Hydrodynamic sizes and polydispersity indices (PDI) of R_8_L_10_@CAT and CR_8_L_10_@CAT prepared at different peptide: CAT weight ratios (15:1, 10:1, 5:1, and 1:1), as determined by DLS. (C) Experimental design for evaluating bladder retention after intravesical instillation by in *vivo* imaging system (IVIS) and confocal laser scanning microscopy (CLSM). (D) Ex vivo IVIS images and quantification of mouse bladders 24 h after intravesical instillation of Cy5.5‐labeled CAT (CAT‐Cy5.5), R_8_L_10_@CAT‐Cy5.5, or CR_8_L_10_@CAT‐Cy5.5 prepared at the different peptide: CAT weight ratios. (E) CLSM images and fluorescence intensity quantification of bladder sections collected 24 h after instillation of CAT‐Cy5.5, R_8_L_10_@CAT‐Cy5.5, or CR_8_L_10_@CAT‐Cy5.5 (10:1) (scale bar: 200 µm). (F) TEM micrograph of CR_8_L_10_@CAT (10:1) and DLS size distributions of free CAT and CR_8_L_10_@CAT (10:1) (scale bar: 200 nm). (G) Representative HAADF‐STEM images of AuNPs incubated with R_8_L_10_@CAT (top left) (scale bar: 100 nm) or CR_8_L_10_@CAT (top right). Hydrodynamic diameters of R_8_L_10_@CAT and CR_8_L_10_@CAT before (−) and after (+) incubation with AuNPs, measured by DLS (bottom). (H) Photographs of CR_8_L_10_@CAT (10:1) as a reconstituted solution and as a lyophilized powder. (I) Relative enzyme activities of CAT and relative surface thiol contents of CR_8_L_10_ in freshly prepared (FS) and reconstituted (RS) CR_8_L_10_@CAT formulations. Data are presented as mean ± SD with n = 3 biological replicates per group. Statistical analysis was performed using one‐way ANOVA with Tukey's multiple comparisons test; ns, not significant; *p < 0.05, **p < 0.01, ***p < 0.001.

Next, to evaluate mucosal retention under physiological clearance conditions, we conducted experiments using the mouse bladder via intravesical instillation, a clinical procedure commonly employed for the treatment of bladder diseases. CAT was labeled with Cy5.5 (CAT‐Cy5.5) and subsequently self‐assembled with R_8_L_10_ and CR_8_L_10_, forming R_8_L_10_@CAT‐Cy5.5 and CR_8_L_10_@CAT‐Cy5.5 nanocomplexes, respectively. Each formulation was instilled into the mouse bladders and retained for 2 h, followed by free voiding over a 24‐hour period (Figure 1C). Then, bladders were harvested for in vivo imaging system (IVIS). IVIS and the corresponding fluorescence quantification revealed that R_8_L_10_@CAT showed a stronger signal than free CAT, consistent with enhanced mucoadhesion mediated by electrostatic interactions between the cationic carrier and the negatively charged mucosa. Notably, at all peptide: CAT ratios tested, CR_8_L_10_@CAT exhibited higher bladder fluorescence than the corresponding R_8_L_10_@CAT formulations, and the 10:1 CR_8_L_10_@CAT group showed the strongest residual signal (Figure 1C, D), demonstrating more robust bladder retention against urine flushing. We hypothesize that the smaller particle size, which corresponds to a larger specific surface area, would enhance mucoadhesion by providing more interaction sites for binding to the mucosal layer. The harvested bladders were further processed into frozen sections and analyzed by confocal laser scanning microscopy (CLSM). In Figure 1E, the CR_8_L_10_@CAT‐Cy5.5 formulation at a 10:1 peptide‐to‐CAT ratio exhibited markedly stronger fluorescence intensity than other groups, and the fluorescence was primarily localized around the mucosa (Figure 1E). Therefore, the CR_8_L_10_:CAT weight ratio of 10:1 was selected as the optimal formulation for subsequent experiments.

Next, we carefully characterized CR_8_L_10_@CAT nanoparticles prepared at the optimal ratio. Transmission electron microscopy (TEM) imaging showed a uniform nanoparticle morphology, and DLS size‐distribution analysis showed a narrow peak centered around 172.4 nm for CR_8_L_10_@CAT (Figure 1F). Free CAT displayed a negative zeta potential of approximately −8.0 mV, whereas CR_8_L_10_@CAT exhibited a positive zeta potential of approximately +8.9 mV after co‐assembly (Figure S1). The loading efficiency of CAT was quantified by an ultrafiltration‐based separation method followed by a CAT activity assay. After nanocomplex preparation, the formulation was subjected to ultrafiltration to separate free CAT from nanocomplex‐associated CAT, and the CAT activity in the filtrate was measured to quantify the unbound fraction. Free CAT was nearly undetectable in the ultrafiltrate, indicating that nearly all CAT was associated with the nanocomplexes. It is well established that thiol groups can coordinate with gold nanoparticles (AuNPs), forming Au‐S bonds [25]. This property provides a widely used approach for detecting the exposure of reactive thiol residues. To assess the presence of accessible thiol groups on the nanocomplexes, we incubated R_8_L_10_@CAT and CR_8_L_10_@CAT with AuNPs (≈5 nm in diameter), respectively. High‐angle annular dark‐field‐scanning TEM (HAADF‐STEM) images showed that AuNPs were densely and uniformly distributed on CR_8_L_10_@CAT, whereas negligible binding was observed with R_8_L_10_@CAT. Consistently, DLS measurements demonstrated an increase in the hydrodynamic diameter of CR_8_L_10_@CAT by approximately 12.8 nm following incubation with AuNPs, which is in agreement with the formation of a monolayer of AuNPs on the nanocomplex surface. In contrast, the size of R_8_L_10_@CAT remained unchanged. Collectively, these results confirm the presence of abundant reactive thiol groups on the surface of CR_8_L_10_@CAT nanocomplexes (Figure 1G).

Next, we examined the dilution stability of CR_8_L_10_@CAT over a broad concentration range. Across a wide concentration range of CR_8_L_10_ (0.036‐10 mg/mL), the hydrodynamic diameter of CR_8_L_10_@CAT remained essentially unchanged, indicating good dilution stability (Figure S2). To assess the influence of the self‐assembly process on the enzyme activity, we compared the enzyme activity of CAT and CR_8_L_10_@CAT by monitoring H_2_O_2_ decomposition kinetics. The results showed that CR_8_L_10_@CAT retained enzymatic activity comparable to that of CAT, and the H_2_O_2_ consumption curves of the two formulations almost overlapped, suggesting that the non‐covalent interactions involved in the self‐assembly process exerted a negligible impact on the CAT enzyme activity (Figure S3). Furthermore, the stability of CR_8_L_10_@CAT was investigated by incubation in artificial urine at 37°C for up to 48 h. CR_8_L_10_@CAT showed negligible changes in hydrodynamic diameter and maintained its CAT activity throughout the incubation period, demonstrating its excellent colloidal and functional stability in urine conditions (Figure S4). In addition, we evaluated the stability of CR_8_L_10_@CAT in 10% FBS‐containing medium to simulate protein‐rich biological environments. Similarly, the nanocomplexes remained stable with minimal changes in their hydrodynamic diameters and CAT activity in the presence of serum proteins (Figure S5). Considering the formulation requirements for clinical translation, we further evaluated the feasibility of preparing CR_8_L_10_@CAT as a lyophilized product. The nanocomplex could be readily freeze‐dried into a loose, free‐flowing powder and rapidly reconstituted into a clear, homogeneous solution (Figure 1H). Importantly, both the CAT enzymatic activity and the surface thiol content of CR_8_L_10_ were well preserved after lyophilization and reconstitution, with no significant differences between freshly prepared (FS) and reconstituted (RS) samples (Figure 1I). In addition to CAT, CR_8_L_10_ was capable of self‐assembling with different types of proteins, including superoxide dismutase (SOD, 32 kDa), bovine serum albumin (BSA, 66 kDa), and immunoglobulin G (IgG, 150 kDa), forming uniform nanoparticles. These results demonstrated that CR_8_L_10_ serves as a versatile platform for delivering proteins with diverse molecular weights, highlighting its broad applicability in biomolecule‐based therapeutic strategies (Figure S6).

### 2.2 Mucoadhesion Mechanism of CR_8_L_10_@CAT

Given the abundance of accessible thiols on CR_8_L_10_@CAT, we hypothesized that these thiols contribute to its mucoadhesive behavior. To test this hypothesis, we first investigated the relationship between surface thiol content and mucoadhesive capacity. We employed N‐ethylmaleimide (NEM) to partially block the free thiols on CR_8_L_10_@CAT via a thiol–maleimide Michael addition reaction. The mucin‐binding capability of NEM‐treated CR_8_L_10_@CAT was then evaluated using mucin‐coated enzyme‐linked immunosorbent assay (ELISA) plates by quantifying the plate‐retained CAT [16]. Meanwhile, the residual thiol content of NEM‐treated‐CR_8_L_10_@CAT was quantified by Ellman's assay (DTNB). As shown in Figure 2A, a plot of plate‐retained CAT activity versus residual free‐thiol content revealed a positive dose‐dependent correlation, directly linking thiol availability to mucoadhesive performance. These results support that free thiols are required for the enhanced mucin binding of CR_8_L_10_@CAT, consistent with a thiol‐disulfide exchange‐mediated mucoadhesion mechanism (Figure 2A).

**FIGURE 2:**
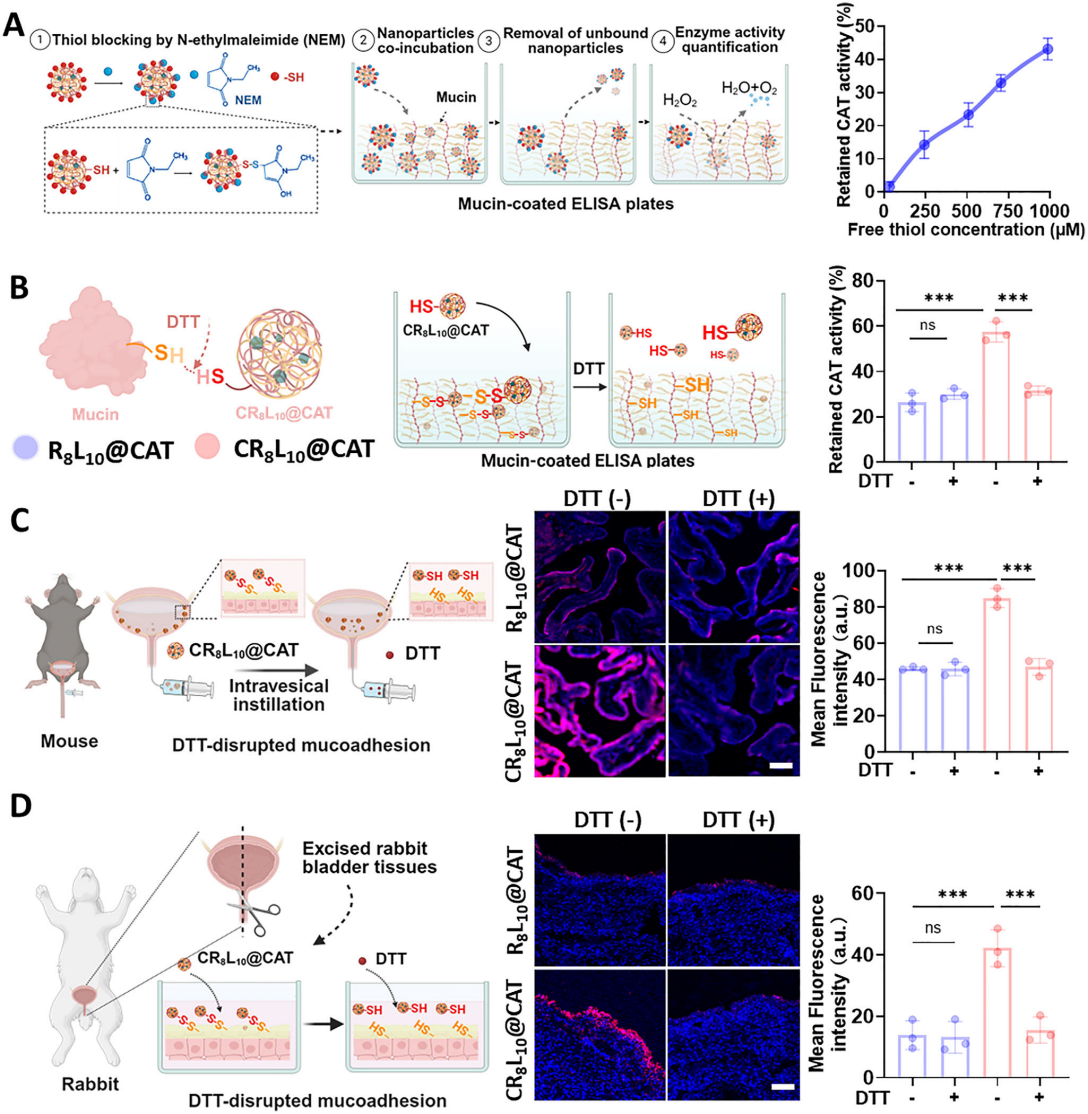
Mucoadhesion mechanism of CR_8_L_10_@CAT. (A) Schematic illustration of tuning residual surface thiols on CR_8_L_10_@CAT by N‐ethylmaleimide (NEM) capping, generating a gradient of available thiol groups and corresponding mucin‐binding capacity (left). The plot shows the correlation between residual free thiols and mucin‐retained catalase activity after incubation on mucin‐coated plates (right). (B) Experimental verification of disulfide‐bond anchoring between CR_8_L_10_@CAT and mucin. Schematic of thiol‐disulfide exchange between mucin‐coated plates and CR_8_L_10_@CAT (left); quantification of mucin‐retained CAT activity following incubation of CR_8_L_10_@CAT or R_8_L_10_@CAT with mucin‐coated plates in the presence or absence of DTT (right). (C, D) CLSM images of bladder tissues showing mucosal adhesion of R_8_L_10_@CAT‐Cy5.5 and CR_8_L_10_@CAT‐Cy5.5 in the presence or absence of DTT. (C) Schematic illustration (left) and CLSM images (right) of the mouse bladder sections after intravesical instillation for 2 h (scale bar: 100 µm). (D) Schematic illustration (left) and CLSM images (right) of the rabbit bladder sections after ex vivo incubation for 2 h. Nuclei were counterstained with DAPI (blue) (scale bar: 100 µm). Data are presented as means ± SD with n = 3 biological replicates per group. Statistical analysis was performed using one‐way ANOVA with Tukey's post‐hoc test; ns, not significant; *p < 0.05, **p < 0.01, ***p < 0.001.

Given the fact that mucins on mucosal surfaces are enriched with disulfide bonds, we hypothesized that the enhanced mucoadhesion of CR_8_L_10_@CAT is mediated by dynamic thiol‐disulfide exchange between surface cysteine residues and disulfide linkages within mucins. To test this hypothesis, mucin‑coated ELISA plates were incubated with R_8_L_10_@CAT or CR_8_L_10_@CAT in the presence or absence of the reducing agent dithiothreitol (DTT). CAT activity retained on mucin‑coated ELISA plates was measured as an indirect indicator of mucoadhesion mediated by thiol‑disulfide exchange. In the absence of DTT, CR_8_L_10_@CAT exhibited substantially higher enzyme retention than R_8_L_10_@CAT, indicating the stronger interaction of CR_8_L_10_@CAT with mucin. In the presence of DTT, CR_8_L_10_@CAT retention decreased markedly compared with the DTT‐free condition, supporting a thiol‐disulfide exchange‐mediated adhesion mechanism (Figure 2B).

To obtain more direct evidence for disulfide bonds between CR_8_L_10_@CAT and mucin, CR_8_L_10_@CAT was first co‐incubated with mucin. The remaining free thiols in the mixture were then blocked with NEM via thiol‐maleimide conjugation. The samples were subsequently treated with tris (2‐carboxyethyl) phosphine (TCEP), a disulfide‐reducing reagent. Then, the resulting samples were labeled with Cy3‐maleimide, followed by ultrafiltration to remove unbound Cy3‐maleimide. Finally, the fluorescence signal was measured to reflect thiol sites exposed after reduction of disulfide bonds. As shown in Figure S7, a markedly stronger Cy3 signal was observed in the TCEP‐treated group than in the untreated group. This result supports the formation of disulfide bonds between CR_8_L_10_@CAT and mucin (Figure S7).

Then, we investigated the adhesion mechanism in vivo. R_8_L_10_@CAT‐Cy5.5 or CR_8_L_10_@CAT‐Cy5.5 was instilled intravesically into mouse bladders and retained for 2 h. Subsequently, bladders were exposed to either DTT or phosphate‐buffered saline (PBS) for 30 min, followed by thorough rinsing with PBS before being harvested for cryo‐sectioning. Under DTT treatment, the mucosal fluorescence signals of CR_8_L_10_@CAT‐Cy5.5‐treated bladders decreased significantly, while those in R_8_L_10_@CAT‐Cy5.5‐treated bladders remained nearly unchanged (Figure 2C). This finding further demonstrated that the disulfide bond formed between cysteine residues on the CR_8_L_10_@CAT surface and the disulfide linkages in mucins is likely responsible for the prolonged bladder retention of CAT. To evaluate cross‐species generalizability, freshly excised rabbit bladders were incubated with CR_8_L_10_@CAT‐Cy5.5 for 2 h in the presence or absence of DTT, followed by washing with artificial urine. DTT markedly diminished the mucosal fluorescence signals of CR_8_L_10_@CAT‐Cy5.5, indicating that reducing conditions effectively disrupt CR_8_L_10_‐mediated mucoadhesion (Figure 2D). These findings indicated that the thiol‐disulfide exchange mechanism underlying CR_8_L_10_@CAT retention is conserved across species.

### 2.3 Antioxidant and Anti‐Inflammatory Effects of CR_8_L_10_@CAT In Vitro

Effective scavenging of ROS at inflamed sites is vital to prevent oxidative tissue injury and inhibit inflammatory responses. To assess the ROS‐scavenging capacity of CR_8_L_10_@CAT in vitro, RAW264.7 macrophages were stimulated with H_2_O_2_ (200 µM) and co‐incubated for 12 h with PBS, free CAT, or CR_8_L_10_@CAT. Intracellular ROS levels were then stained using the fluorescent probe 2′,7′‐dichlorodihydrofluorescein diacetate (DCFH‐DA) and analyzed by confocal microscopy and flow cytometry [26]. Confocal imaging showed that H_2_O_2_ stimulation markedly elevated ROS‐associated fluorescence, whereas both CAT and CR_8_L_10_@CAT restored fluorescence to near‐baseline levels. Flow cytometry showed that the proportion of ROS‐positive cells decreased from 26.4% in H_2_O_2_‐stimulated controls to 5.6% with CAT treatment and 4.7% with CR_8_L_10_@CAT treatment, respectively (Figure S8). These findings demonstrated the potent ROS‐scavenging capability of CAT and CR_8_L_10_@CAT. We further evaluated the antioxidant activity using a lipopolysaccharide (LPS)‐stimulated inflammatory model, which induces cellular oxidative stress by activating redox‐sensitive signaling pathways [10]. For CLSM observations, the LPS‐treated group exhibited stronger fluorescence, confirming the successful induction of oxidative stress by LPS stimulation. Compared with the LPS‐treated group, ‘LPS + CR_8_L_10_@CAT’ group showed markedly reduced fluorescence signals (Figure 3A), and flow cytometry demonstrated a pronounced reduction in ROS levels following treatment with CR_8_L_10_@CAT (Figure 3B). These results demonstrated the potent antioxidant properties of CR_8_L_10_@CAT in vitro.

**FIGURE 3:**
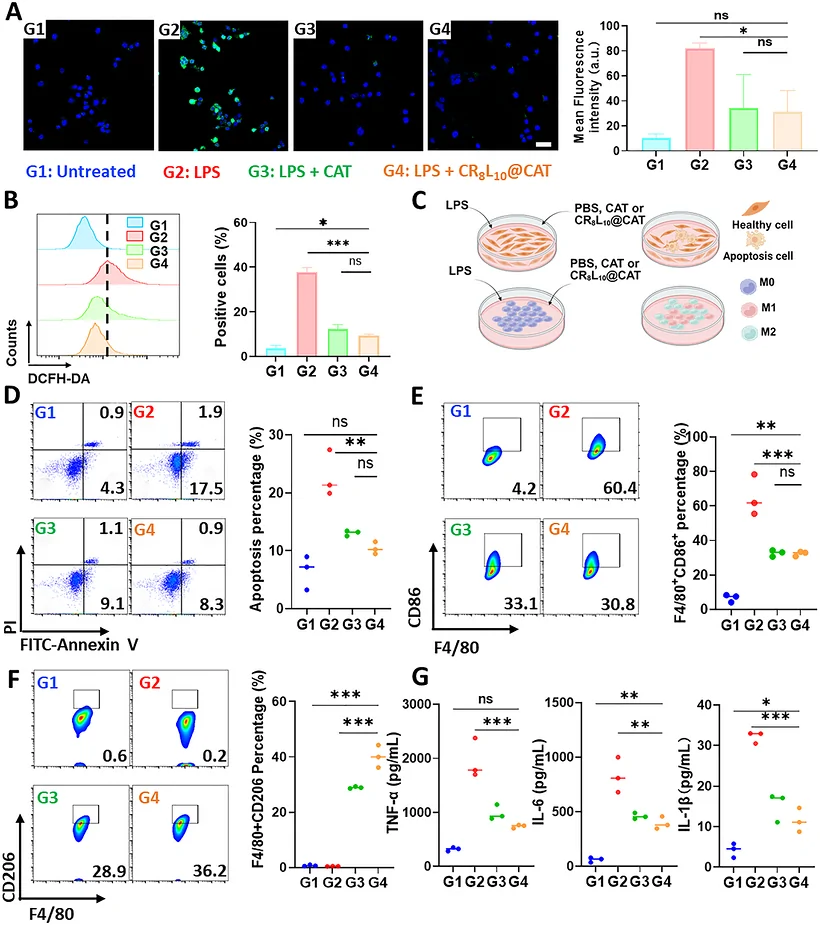
Antioxidant and anti‐inflammatory effects of CR_8_L_10_@CAT in vitro. RAW264.7 macrophages were used for ROS, macrophage polarization, and cytokine assays, while MLE‐12 epithelial cells were used for apoptosis analysis. Groups: untreated (G1), LPS (G2), LPS + CAT (G3), LPS + CR_8_L_10_@CAT (G4). (A) CLSM images and quantification of intracellular ROS in RAW264.7 stained with Hoechst (nuclei, blue) and DCFH‐DA (ROS, green) (scale bar: 20 µm). (B) Flow‐cytometric analysis of intracellular ROS in RAW264.7 cells using DCFH‐DA. (C) Schematic illustration of the in vitro LPS‐induced inflammatory model and the corresponding treatment groups. (D) Apoptosis analysis in MLE‐12 by Annexin V‐FITC/PI staining. (E) Flow‐cytometric quantification of M1 (F4/80^+^CD86^+^) macrophages for RAW264.7 cells. (F) Flow‐cytometric quantification of M2 (F4/80^+^CD206^+^) macrophages for RAW264.7 cells. (G) Cytokine levels of TNF‐α, IL‐6, and IL‐1β produced by RAW264.7 cells were measured by ELISA. Data are presented as means ± SD with n = 3 biological replicates per group. Statistical analysis was performed using one‐way ANOVA with Tukey's post‐hoc test; ns, not significant; *p < 0.05, **p < 0.01, ***p < 0.001.

We next evaluated the anti‐apoptotic effects of nanoantioxidant CR_8_L_10_@CAT in three epithelial cell types: MLE‐12 mouse alveolar epithelial cells, BEAS‐2B human airway epithelial cells, and MB49 mouse bladder epithelial cells. These epithelial cells were stimulated with LPS and then co‐incubated with either CAT or CR_8_L_10_@CAT, followed by Annexin V and propidium iodide (PI) staining to assess apoptosis levels [27]. Treatment with ‘LPS + CR_8_L_10_@CAT’ significantly reduced LPS‐induced apoptosis in three types of epithelial cells. Specifically, in MLE‐12 cells, LPS stimulation increased the apoptotic rate to 19.4%, compared with 5.2% in the untreated group. This rate was reduced to 10.2% in the ‘LPS + CAT’ group and decreased to 9.1% in the ‘LPS + CR_8_L_10_@CAT’ group. Similar trends were observed in MB49 and BEAS‐2B cells. These results suggest that CAT and CR_8_L_10_@CAT were able to effectively protect epithelial cells from oxidative stress (Figure 3D, Figure S9).

M1 macrophages drive inflammatory responses by producing ROS and secreting pro‐inflammatory cytokines, whereas M2 macrophages contribute to inflammation resolution and tissue repair by releasing anti‐inflammatory mediators [28, 29]. Thus, strategies aiming at reprogramming macrophages from the pro‐inflammatory M1 phenotype to the anti‐inflammatory M2 phenotype hold great promise for anti‐inflammatory therapies. To evaluate macrophage polarization and the anti‐inflammatory properties of CR_8_L_10_@CAT, RAW264.7 cells were co‐treated with LPS and either PBS, CAT, or CR_8_L_10_@CAT. With LPS treatment, M1 macrophages (F4/80^+^CD86^+^) increased to approximately 60.4%, and M2 macrophages (F4/80^+^CD206^+^) decreased to approximately 0.2%, indicating a shift toward a pro‐inflammatory state. Notably, treatment with ‘LPS + CAT’ or ‘LPS + CR_8_L_10_@CAT’ reduced the percentages of M1 macrophages to 33.1% or 30.8%, and increased the percentages of M2 macrophages to 28.9% or 36.2%, respectively (Figure 3E, F). Pro‐inflammatory cytokines play a crucial role in sustaining and amplifying inflammatory responses [30]. We quantified the levels of tumor necrosis factor‐alpha (TNF‐α), interleukin‐6 (IL‐6), and interleukin‐1β (IL‐1β) in cell supernatants using ELISA. As expected, compared with the LPS‐treated group, the levels of these inflammatory cytokines were significantly reduced in both ‘LPS + CAT’ and ‘LPS + CR_8_L_10_@CAT’ groups, decreasing to levels approaching the baseline observed in the untreated group (Figure 3G). These results demonstrate that our CR_8_L_10_@CAT nanoantioxidant not only drives macrophage repolarization from the pro‐inflammatory M1 phenotype to the anti‐inflammatory M2 phenotype, but also potently inhibits the release of key pro‐inflammatory cytokines, collectively underscoring its strong potential for anti‐inflammatory therapy.

### 2.4 Intravesical Instillation of CR_8_L_10_@CAT for Treatment of IC/BPS

To evaluate the bladder exposure profile of CAT, we quantified active CAT levels in bladder tissue at 2, 6, 12, 24, and 48 h post‐treatment. Mice were intravesically administered free CAT or CR_8_L_10_@CAT at a CAT‐equivalent dose (1 mg/mL, 50 µL) and retained in the bladder for 2 h. Then, the bladders were collected at the indicated time points and CAT levels were quantified using a commercial CAT assay kit. As shown in Figure S10, the CAT concentration in the CR_8_L_10_@CAT group was approximately 1.4, 1.8, 2.0, 2.6, and 2.8 times that in the free CAT group at 2, 6, 12, 24, and 48 h, respectively (Figure S10). Compared with CAT, CR_8_L_10_@CAT achieved more sustained local retention against urinary washout by mucoadhesion. At 24 h, the concentration of CAT in non‐target organs (heart, liver, spleen, lung, and kidney) remained at low levels for both free CAT and CR_8_L_10_@CAT, indicating limited systemic exposure and minimal off‐target exposure following bladder dosing (Figure S11). Together, these data indicate that CR_8_L_10_@CAT enhances local bladder exposure while limiting systemic leakage, providing a pharmacokinetic basis for improved efficacy and systemic safety.

Encouraged by the anti‐inflammatory performance in vitro and prolonged bladder retention of CR_8_L_10_@CAT nanoantioxidant, we then investigated its potential as an intravesical anti‐inflammatory therapy to treat chronic interstitial cystitis/bladder pain syndrome (IC/BPS). A chronic IC/BPS mouse model was established using an LPS/PS intravesical instillation regimen as reported previously [2]. Briefly, protamine sulfate (PS, 30 mg/mL) was first intravesically instilled to transiently disrupt the urothelial barrier, followed by intravesical instillation of LPS (4 mg/mL) to induce bladder inflammation, according to the schedule depicted in Figure 4A. We first conducted a dose‐dependent therapeutic study in the IC/BPS model. Treatment with 0.25 mg/mL CR_8_L_10_@CAT produced only a modest reduction in TNF‐α, IL‐1β, and IL‐6, whereas 0.5 mg/mL led to a more evident suppression of these inflammatory cytokines. In contrast, 1 mg/mL CR_8_L_10_@CAT produced the most pronounced anti‐inflammatory effect, reducing all three cytokines to levels approaching those of healthy mice. To further evaluate the therapeutic advantage of CR_8_L_10_@CAT in the IC/BPS model, we compared it with the non‐adhesive control, free CAT, and the cationic control, R_8_L_10_@CAT. As shown in Figure S12, untreated IC/BPS mice displayed markedly increased levels of pro‐inflammatory cytokines, including TNF‐α, IL‐1β, and IL‐6, compared with healthy mice. Relative to the untreated group, free CAT and R_8_L_10_@CAT produced only limited cytokine suppression; CR_8_L_10_@CAT achieved a more effective anti‐inflammatory effect (Figure S12). Based on these results, 1 mg/mL was selected for subsequent intravesical treatment studies.

**FIGURE 4:**
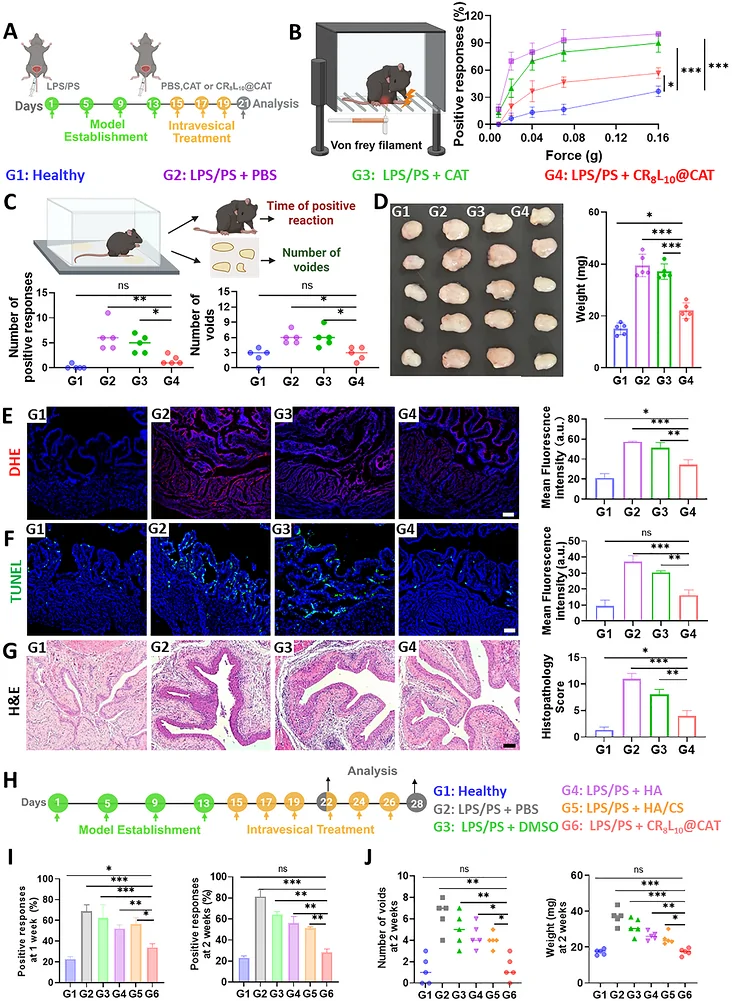
CR_8_L_10_@CAT for treatment of interstitial cystitis/bladder pain syndrome (IC/BPS). (A–G) LPS/PS‐induced IC/BPS mice were intravesically instilled with PBS, free CAT, or CR_8_L_10_@CAT (CAT‐equivalent dose, 1 mg/mL) every other day for three times, with a healthy group included as control. Groups: Healthy (G1), LPS/PS + PBS (G2), LPS/PS + CAT (G3), LPS/PS + CR_8_L_10_@CAT (G4). (A) Schematic of model establishment and intravesical therapy schedule. (B) Stimulus‐evoked mechanical allodynia assessed by graded von Frey filaments applied to the suprapubic region, with stimulus–response curves plotted against filament force. (C) Spontaneous nociceptive behaviors (licking/guarding) were recorded over 15 min, and voiding frequencies were measured in metabolic cages over 2 h. (D) Representative bladder morphology and quantification of bladder wet weights. (E) CLSM images and quantification of ROS in bladder sections stained with DHE (scale bar: 100 µm). (F) CLSM images and quantification of apoptosis in bladder sections stained with TUNEL (scale bar: 100 µm). (G) H&E‐stained bladder sections with histopathological scoring (scale bar: 100 µm). (H) Comparison of intravesical CR_8_L_10_@CAT with clinically used intravesical agents. LPS/PS‐induced IC/BPS mice were treated with PBS, DMSO, HA, HA/CS or CR_8_L_10_@CAT on the same intravesical schedule, with a healthy group as control (groups: Healthy, IC/BPS + PBS, IC/BPS + DMSO, IC/BPS + HA, IC/BPS + HA/CS, IC/BPS + CR_8_L_10_@CAT). (I) Positive response rates at 0.16 g von Frey stimulation after 1 and 2 weeks of treatment. (J) Voiding frequencies and bladder wet weights at the end of the 2‐week treatment period. All data are presented as mean ± SD (n = 5). Statistical analysis was performed using one‐way ANOVA with Tukey's post‐hoc test; ns, not significant; *p < 0.05, **p < 0.01, ***p < 0.001.

Following IC/BPS model establishment, mice were then treated intravesically with PBS, CAT, or CR_8_L_10_@CAT every other day for three administrations at an equal CAT‐equivalent dose of 1 mg/mL (50 µL). Therapeutic effects were evaluated 48 h after the final instillation. Initially, we measured pain‐related behavioral responses and voiding functions in the IC/BPS mice (Figure 4A). Pain‐related behavioral responses to mechanical stimulation were quantified using graded von Frey filaments applied to the lower abdominal region [31]. A positive response was defined as licking or guarding of the stimulated site, abdominal withdrawal, or escape/jumping behaviors. Stimulus‐response curves were generated by plotting the proportion of positive responses against the corresponding filament force. Compared with healthy controls, the PBS‐treated model mice exhibited significantly elevated response rates across all force intensities, confirming successful induction of the IC/BPS model with heightened pain sensitivity. In contrast to the PBS group, the free CAT group showed a modest attenuation in pain responses, and the CR_8_L_10_@CAT group exhibited a marked downward shift in the stimulus‐response curve. Specifically, at the highest filament force (0.16 g), the response rate in the CR_8_L_10_@CAT group was approximately 44.0% lower than that in the PBS group and 37.8% lower than that in the CAT group, demonstrating statistically significant alleviation of bladder pain‐related behavior (Figure 4B). We subsequently assessed spontaneous pain behaviors by recording the number of predefined pain‐related responses in the absence of external stimulation. Mice instilled with CR_8_L_10_@CAT showed a marked reduction in licking of the abdomen or urethra compared with the PBS group, suggesting effective relief of spontaneous pain. To evaluate voiding functions, voiding frequency was measured in metabolic cages by counting void spots on filter paper [32]. CR_8_L_10_@CAT treatment reduced voiding frequency to a level comparable to that of the healthy group, indicating effective attenuation of bladder overactivity (Figure 4C). The behavior studies demonstrated that the intravesically applied CR_8_L_10_@CAT remarkably improved bladder functions in IC/BPS mice.

As inflammatory edema is a major contributor to bladder wall thickening and dysfunction in IC/BPS, we collected bladder tissues to observe morphological changes and quantified their wet weights to evaluate this critical aspect of disease pathology. Severe inflammatory edema was observed in the PBS group, as evidenced by marked bladder distension, hyperemia, and a significant increase in the average wet weight from 15.1 mg in healthy controls to 39.5 mg in the PBS‐treated model group. Compared with the PBS group, the CAT group showed only a modest alleviation of edema, as evidenced by a mild morphological improvement and a limited reduction in bladder wet weights. In contrast, CR_8_L_10_@CAT treatment conferred a robust protective effect, as reflected by the marked resolution of bladder distension and hyperemia, as well as a pronounced decrease in the average wet weight of bladders to 22.1 mg, which corresponds to reductions of 44.1% and 40.6% relative to the PBS and CAT groups, respectively. Our results collectively demonstrate the superior capacity of CR_8_L_10_@CAT to mitigate bladder edema (Figure 4D).

Next, we validated the therapeutic effects at the histological level. Bladder sections were stained with the ROS‐sensitive fluorescent probe dihydroethidium (DHE). Notably, CR_8_L_10_@CAT treatment effectively reduced the ROS level in the bladder tissue to a level comparable to healthy controls, demonstrating its strong ROS‐scavenging activity (Figure 4E). We subsequently assessed apoptosis levels on bladder sections using the terminal deoxynucleotidyl transferase‐mediated dUTP nick‐end labeling (TUNEL) assay, and the fluorescent signals were quantified. As expected, the apoptotic signals in bladder tissues were markedly reduced by CR_8_L_10_@CAT treatment, highlighting its protective performance. In mice with IC/BPS, excessive ROS and the ensuing oxidative stress in bladder tissue drive urothelial injury and amplify inflammatory responses. (Figure 4F). Therefore, we evaluated tissue integrity by hematoxylin and eosin (H&E) staining. Representative H&E sections from the PBS‐treated model group showed marked mucosal thickening and extensive inflammatory‐cell infiltration compared with the healthy group. In contrast, CR_8_L_10_@CAT group exhibited restored mucosal thickness with orderly folds and minimal inflammatory‐cell infiltration, indicating effective attenuation of inflammation‐induced tissue damage (Figure 4G). Our results thus demonstrate that mucoadhesive CR_8_L_10_@CAT upon intravesical instillation could efficiently scavenge ROS, protect bladder tissues from oxidative damage, restore tissue architecture, and preserve bladder functions, leading to effective treatment of IC/BPS.

To further confirm the therapeutic efficacy of CR_8_L_10_@CAT, we included the clinical standard treatments using dimethyl sulfoxide (DMSO), hyaluronic acid (HA), or a hyaluronic acid/chondroitin sulfate combination (HA/CS). The therapeutic effects of DMSO are generally attributed to its osmotic activity and nonspecific anti‐inflammatory and analgesic properties, which help alleviate bladder pain and urinary frequency [33, 34]. HA and HA/CS are glycosaminoglycan (GAG)‐replacement formulations that primarily function by protecting and restoring the bladder mucosal barrier [35, 36]. Following 1 and 2 weeks of repeated intravesical instillation of PBS, DMSO, HA, HA/CS, or CR_8_L_10_@CAT, we assessed pain‐related behavioral responses of IC/BPS model mice in each group using von Frey mechanical stimulation. Overall, all treatment groups exhibited reduced positive response rates compared with the PBS group at both 1 and 2 weeks (Figure 4H). Notably, the CR_8_L_10_@CAT group showed the most pronounced downward shift of the stimulus‐response curve, which nearly overlapped with that of healthy controls after 2 weeks of treatment, suggesting a faster onset and better persistence of analgesic efficacy than the clinically used intravesical agents (Figure S13). Under high‐intensity stimulation (0.16 g), we further quantified the extent of improvement relative to the PBS group. After 1 week of treatment, the positive response rates in the DMSO, HA, and HA/CS groups decreased by approximately 9.0%, 27.8%, and 18.3%, respectively, whereas the CR_8_L_10_@CAT group showed a reduction of about 51.0%, clearly outperforming the three clinically used intravesical agents. After 2 weeks of treatment, this trend became even more pronounced: the positive response rates in the DMSO, HA, and HA/CS groups decreased by approximately 21.6%, 31.3%, and 37.4% relative to PBS, but still remained substantially higher than those of healthy mice. In contrast, the CR_8_L_10_@CAT group exhibited an approximately 65% reduction compared with PBS and showed the smallest deviation from healthy controls (Figure 4I).

Regarding voiding function, bladder hyperactivity was evaluated 2 weeks after the start of treatment by quantifying void spots on filter paper using a void spot assay (Figure 4J). After treatment, DMSO, HA, and HA/CS slightly reduced the markedly elevated voiding frequency in IC/BPS mice, but the values remained higher than those of healthy mice, indicating only partial improvement. By contrast, the CR_8_L_10_@CAT group already showed the most pronounced decrease in voiding frequency at this time point, with values approaching normal levels. Subsequently, at the end of the 2‐week treatment period, bladder tissues were harvested, and bladder wet weights were measured to assess inflammatory edema. The PBS group exhibited a significant increase in the average bladder wet weight, indicating pronounced bladder wall edema. DMSO, HA, and HA/CS partially reduced bladder wet weights, but their values remained higher than those in healthy mice, reflecting only limited resolution of edema. In contrast, the CR_8_L_10_@CAT group showed a much greater reduction in the average bladder wet weight, approaching the healthy range, indicating superior protection against inflammatory edema and structural alterations of the bladder wall (Figure 4J; Figure S14). Our results clearly demonstrated that CR_8_L_10_@CAT would be a more potent therapeutic agent compared with other intravesical agents currently used in clinic for IC/BPS treatment.

To evaluate the systemic safety of CR_8_L_10_@CAT after intravesical administration, we measured serum biochemistry parameters and hematological indices in mice treated with CR_8_L_10_@CAT at 1 mg/mL (therapeutic dose) or 2 mg/mL (twofold therapeutic dose). As shown in Figure S15, liver‐ and kidney‐related biochemical markers, including alanine aminotransferase (ALT), aspartate aminotransferase (AST), alkaline phosphatase (ALP), and blood urea nitrogen (BUN), remained within a comparable range across the healthy, 1 mg/mL, and 2 mg/mL groups. In addition, basic hematology indices, including white blood cell count (WBC), red blood cell count (RBC), hemoglobin (HGB), hematocrit (HCT), mean corpuscular volume (MCV), mean corpuscular hemoglobin (MCH), mean corpuscular hemoglobin concentration (MCHC), and platelet count (PLT), showed no apparent differences among groups. Together with the low serum CAT exposure after local administration, these findings indicate no evident systemic off‐target effects of CR_8_L_10_@CAT and further strengthen the safety claim for local bladder delivery (Figure S15).

### 2.5 Therapeutic Mechanism of CR_8_L_10_@CAT Nanoantioxidants

In IC/BPS, excessive ROS‐induced mast‐cell degranulation releases algogenic and barrier‐disrupting mediators, directly leading to bladder pain and epithelial barrier disruption. In parallel, oxidative stress drives the recruitment and activation of neutrophils and macrophages, resulting in increased ROS and pro‐inflammatory cytokines that sustain bladder inflammation and tissue injury [37]. Therefore, we quantitatively assessed these cell populations to determine whether ROS scavenging by CR_8_L_10_@CAT could remodel the immune microenvironment. We first assessed mast cell (CD45^+^c‐Kit^+^FcεRIα^+^) infiltration in bladder tissue using flow cytometry. Compared with the healthy group, a significant increase in the proportion of mast cells in the PBS‐treated model group from 2.7% to 12.7% was observed. Notably, CR_8_L_10_@CAT treatment markedly reduced the mast cell level to 2.0%, demonstrating effective suppression of mast cell infiltration (Figure 5A). We next evaluated neutrophil (CD11b^+^Ly6G^+^) and macrophage (CD11b^+^F4/80^+^) populations in bladders. Following CR_8_L_10_@CAT treatment, the populations of neutrophils and macrophages were reduced from 24.1% and 36.0%, to approximately 10.8% and 22.9%, respectively, both of which were comparable to those in the healthy group (Figure 5B, C). Moreover, CR_8_L_10_@CAT treatment increased the M2/M1 macrophage ratio (F4/80^+^CD206^+^/F4/80^+^CD86^+^), indicating their polarization toward an anti‐inflammatory phenotype (Figure 5D). Apart from innate immune cells, adaptive immune cells, particularly T lymphocytes, also mediated pro‐inflammatory immune responses in IC/BPS. Similarly, CR_8_L_10_@CAT treatment reduced the percentage of T cells from 47.7% to 20.9%, approaching the baseline level of healthy controls (Figure 5E).

**FIGURE 5:**
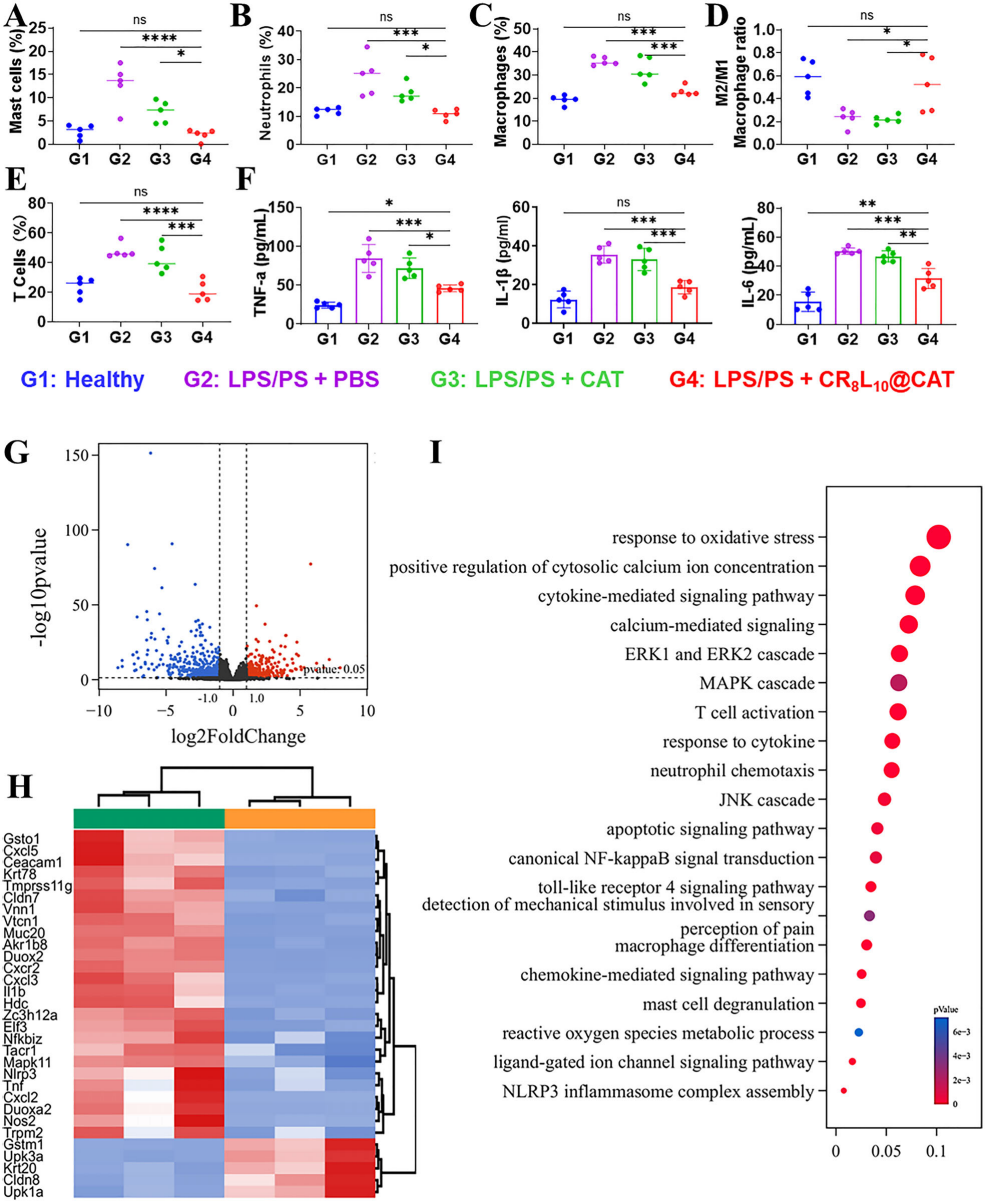
Immune responses and therapeutic mechanism of CR_8_L_10_@CAT. Bladder tissues from treated IC/BPS mice were analyzed by flow cytometry, ELISA, and RNA‐seq. (A–F) Groups: Healthy (G1), LPS/PS + PBS (G2), LPS/PS + CAT (G3), LPS/PS + CR_8_L_10_@CAT (G4); (A–E) Flow‐cytometric quantification of infiltrated immune cells, including (A) Mast cells (CD45^+^c‐Kit^+^FcεRIα^+^); (B) Neutrophils (CD11b^+^Ly6G^+^); (C) Macrophages (CD11b^+^F4/80^+^); (D) M2 (F4/80^+^CD206^+^)/M1 (F4/80^+^CD86^+^) macrophage ratio; and (E) T cells (CD45^+^CD3^+^). (F) Cytokine levels (TNF‐α, IL‐6, and IL‐1β) in bladder homogenates measured by ELISA. (G) Volcano plot of differentially expressed genes (DEGs) in bladder tissues comparing G2 (LPS/PS + PBS) (Left) and G4 (LPS/PS + CR_8_L_10_@CAT) (Right). (H) Heatmap of representative DEGs shown as row‐scaled z‐scores with hierarchical clustering. Samples are arranged with G2 on the left and G4 on the right, and genes are annotated by functional relevance. (I) Gene Ontology enrichment of CR_8_L_10_@CAT‐regulated genes (bubble size: gene ratio; color: −log_10_(p‐value)). Data are presented as mean ± SD. Flow cytometry & ELISA: Groups G1‐G4 (n = 5). RNA‐seq: G2 vs G4 (n = 3). Statistical analysis was performed using one‐way ANOVA with Tukey's post‐hoc test; ns, not significant; *p < 0.05, **p < 0.01, ***p < 0.001.

We next quantified key pro‐inflammatory cytokines in bladder homogenates by ELISA to further evaluate the inflammatory status across different treatment groups (Figure 5F). Compared with healthy mice, IC/BPS model mice treated with PBS exhibited markedly elevated levels of TNF‐α, IL‐6, and IL‐1β, indicating a pronounced inflammatory response in bladder tissue. Free CAT treatment partially reduced the levels of these cytokines. In contrast, CR_8_L_10_@CAT treatment resulted in a pronounced suppression of TNF‐α, IL‐6, and IL‐1β, with cytokine levels approaching those of the healthy group (Figure 5F). The above results taken together indicated that intravesical delivery of mucoadhesive CR_8_L_10_@CAT nanoantioxidant by attenuating ROS‐driven inflammatory cascade could inhibit both immune cell activation and the release of pro‐inflammatory cytokines, thereby restoring bladder immune homeostasis, which is critical for treatment of IC/BPS.

To elucidate the molecular mechanisms underlying the therapeutic efficacy of CR_8_L_10_@CAT nanoantioxidant, we conducted RNA‐seq analysis to reveal the profound and consistent transcriptomic alterations. Principal component analysis (PCA) revealed clear segregation among treatment groups along PC1, which accounted for 66.17% of the variance. The tight within‐group clustering further indicated a consistent and robust reprogramming of the transcriptome (Figure S16). Volcano‐plot analysis (|log_2_FC| ≥ 1, FDR < 0.05) identified 1411 differentially expressed genes (DEGs), including 381 upregulated and 1030 downregulated transcripts, in the CR_8_L_10_@CAT group compared with PBS controls (Figure 5G).

Based on the comprehensive transcriptomic data, CR_8_L_10_@CAT treatment exerted multi‐faceted therapeutic effects through coordinated molecular alterations beyond redox regulation. Volcano plot analysis of differentially expressed genes was conducted to identify transcriptomic alterations in response to CR_8_L_10_@CAT treatment. First, at the level of oxidative stress and lipid peroxidation, CR_8_L_10_@CAT markedly reshaped redox‐related transcriptional programs. The expression of ROS‐generating enzymes, including *Duox2, Duoxa2*, and *Nos2*, was significantly downregulated, consistent with the observed reduction in ROS levels in bladder tissues and suggesting that exogenous CAT, by decomposing H_2_O_2_, decreases upstream ROS burden and limits hydroxyl radical formation. In parallel, core oxidative‐stress regulators such as *Hif1a* and *Nupr1*, as well as the injury‐associated gene *Plat*, were also suppressed, indicating an overall relief of cellular oxidative stress. Conversely, multiple antioxidant and detoxification genes, including *Gstm1*, *Akr1b8*, and *Serpina3n*, were upregulated, reflecting activation of a glutathione‐based detoxification system that facilitates the removal of toxic aldehydes generated by lipid peroxidation. Notably, the glutathione‐synthesis and utilization genes *Slc7a11* and *Gpx2* were concomitantly induced, pointing to a reinforced glutathione‐GPX antioxidant axis that is critical for eliminating phospholipid hydroperoxides and protecting against ferroptosis. Together, these changes indicate that CR_8_L_10_@CAT not only reduces ROS production but also strengthens endogenous antioxidant and detox pathways to restore redox homeostasis.

Concurrently, pain‐associated mediators such as *Trpm2 and Tacr1* were downregulated, a transcriptomic change that aligns with the alleviation of pain‐related behaviors and the normalization of bladder functions as measured in voiding assays. Furthermore, the treatment promoted structural recovery through the upregulated expression of urothelial barrier genes such as tight junction proteins *Cldn7* and *Cldn8*, and the differentiation marker *Krt20*. These genetic improvements corroborated functional and histological recovery, as evidenced by normalized bladder weights, restored tissue architecture in H&E staining, and reduced apoptosis in TUNEL assays. Additionally, suppression of *Hdc*, a mast cell‐specific gene critical for histamine synthesis and degranulation, was accompanied by a significant decrease in mast cell numbers as quantified by flow cytometry. Concurrently, attenuated expression of M1‐associated pro‐inflammatory mediators such as *Il1b* and *Tnf* supported a phenotypic shift in macrophage polarization, which was validated by flow cytometry showing an increased M2/M1 macrophage ratio.

To integrate gene‐level signals into functional pathways, we performed GO enrichment analysis. Antioxidant pathways, such as *response to oxidative stress* and *reactive oxygen species metabolic process*, were significantly enriched, establishing oxidative stress regulation as a central therapeutic axis. In parallel, enrichment of nociceptive pathways, including d*etection of mechanical stimulus involved in sensory perception* and *ligand‐gated ion channel signaling indicated transcriptional modulation of neural sensitization and pain signaling*, which was consistent with the observed attenuation. Epithelial repair pathways, including *calcium‐mediated signaling* and *regulation of cell–cell adhesion*, were enriched, reflecting programs that support tissue restoration and barrier integrity. At the immune level, broad enrichment of inflammation‐ and immunity‐related pathways, including *Toll‐like receptor signaling, NF‐κB cascade, chemokine‐mediated signaling, MAPK/JNK cascade, neutrophil chemotaxis, macrophage differentiation*, and *T‐cell activation*, highlighted extensive remodeling of the inflammatory microenvironment (Figure 5I). Together, these transcriptomic alterations and GO enrichments define a coordinated therapeutic program integrating antioxidant regulation, nociceptive modulation, epithelial repair, and immune reprogramming_‐_that provides the molecular basis for the broad therapeutic efficacy of CR_8_L_10_@CAT in IC/BPS treatment.

To validate the barrier‐restoration pathway suggested by the RNA‐seq results, we examined the expression of the urothelial barrier–related genes Upk1a and Upk3a by quantitative polymerase chain reaction (qPCR). As shown in Figure S17, both Upk1a and Upk3a were significantly increased in the CR_8_L_10_@CAT‐treated group compared with the PBS‐treated IC/BPS group, supporting restoration of bladder epithelial barrier‐related gene expression after treatment (Figure S17). To validate the involvement of NF‐κB signaling in vivo, we further performed western blot analysis of phosphorylated p65 (p‐p65) and total p65 in bladder tissues. As shown in Figure S18, CR_8_L_10_@CAT treatment reduced the p‐p65/p65 ratio compared with the PBS‐treated group, supporting suppression of NF‐κB activation in bladder tissues (Figure S18).

### 2.6 CR_8_L_10_@CAT Nanoantioxidant to Treat LPS‐Induced ALI

Acute lung injury (ALI), a life‐threatening respiratory condition, can be triggered by diverse insults such as sepsis and viral infections. Excessive ROS also plays a central pathogenic role in ALI by inducing alveolar epithelial apoptosis, disrupting endothelial barrier integrity, and activating NF‐κB‐mediated pro‐inflammatory cascades [38]. Therefore, beyond the use of CR_8_L_10_@CAT in treating IC/BPS, we further extended our investigation to explore its potential in addressing other mucosal inflammatory diseases, particularly pulmonary inflammation.

To evaluate the pulmonary exposure profile of CAT following intratracheal pulmonary administration in lung tissue at 2, 6, 12, 24, and 48 h post‐treatment. Mice received free CAT or CR_8_L_10_@CAT via intratracheal aerosolized administration at an equivalent CAT dose (2 mg/mL, 50 µL), and the lungs were collected at the indicated time points for CAT quantification. As shown in Figure S19, the CAT level in the CR_8_L_10_@CAT group was approximately 1.5, 1.7, 2.6, 2.1, and 2.2 times that in the free CAT group at 2, 6, 12, 24, and 48 h, respectively. Overall, the fold change increased from 2 h to 12 h and then remained relatively stable from 24 h to 48 h, highlighting the mucoadhesion‐mediated enhancement of pulmonary retention by CR_8_L_10_@CAT (Figure S19). Similarly, at 24 h after pulmonary administration, the concentration of CAT in peripheral non‐target organs (heart, liver, spleen, and kidney) was consistently low for both formulations (Figure S20). Together, these data suggest that CR_8_L_10_@CAT complexation primarily influences local pulmonary retention, without causing substantial redistribution to other major organs. We further assessed the pulmonary retention of CAT‐Cy5.5 and CR_8_L_10_@CAT‐Cy5.5 via inhalation. Briefly, mice were anesthetized, placed in a supine position, and the formulations were administered by intratracheal nebulized instillation using a small‐animal microsprayer, which generates micro‐droplets of an optimal size (1–10 µm) for uniform pulmonary distribution [39] (Figure 6A). Lungs were harvested 24 h post‐administration to observe the fluorescent signals in lungs. Both IVIS and CLSM imaging revealed significantly higher fluorescence signals in the lung post inhalation of CR_8_L_10_@CAT‐Cy5.5, compared with those in the CAT‐Cy5.5 group. Analysis of lung sections further confirmed that fluorescent signals were predominantly localized along the airway mucosa (Figure 6B, Figure S21). These findings highlight the mucoadhesion‐enhanced pulmonary retention of CR_8_L_10_@CAT‐Cy5.5.

**FIGURE 6:**
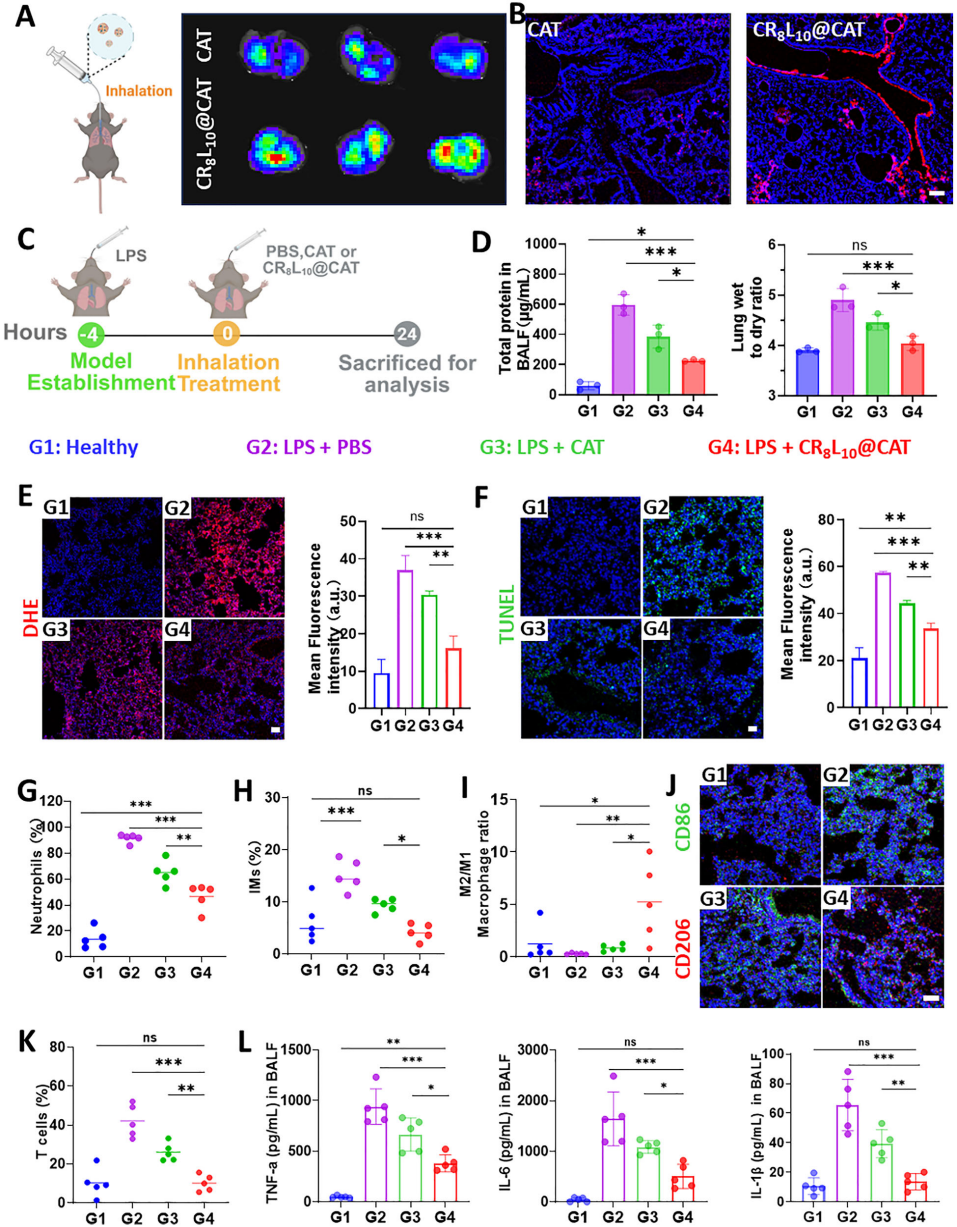
Inhaled CR_8_L_10_@CAT for treatment of acute lung injury (ALI). (A) IVIS imaging of mouse lungs after administration of CAT‐Cy5.5 or CR_8_L_10_@CAT‐Cy5.5. (B) CLSM images of lung sections confirming pulmonary retention and mucosal adhesion of CR_8_L_10_@CAT‐Cy5.5 (scale bar: 100 µm); (C–L) LPS‐induced ALI mice were treated by inhalation of PBS, free CAT, or CR_8_L_10_@CAT (CAT‐equivalent dose, 2 mg/mL), with a healthy group included as a control. Groups: Healthy (G1), LPS + PBS (G2), LPS + CAT (G3), LPS + CR_8_L_10_@CAT (G4); (C) Schematic of the model establishment and inhalation therapy schedule. (D) BALF total protein and lung wet to dry ratios. (E) CLSM images and quantification of ROS in lung sections stained with DHE (scale bar: 20 µm). (F) CLSM images and quantification of apoptosis in lung sections stained with TUNEL (scale bar: 20 µm). (G–J) Flow‐cytometric quantification and CLSM analysis of immune cell infiltration in BALF and lung tissues. (G) Neutrophils (Ly6G^+^CD11b^+^) in BALF. (H) Interstitial macrophages (F4/80^+^CD11b^+^) in lung tissues. (I) M2 (F4/80^+^CD206^+^) to M1 (F4/80^+^CD86^+^) macrophage ratio in lung tissues. (J) CLSM images of lung sections stained for CD86 (M1 marker, green) and CD206 (M2 marker, red) (scale bar: 20 µm). (K) Flow‐cytometric quantification of T cells (CD45^+^CD3^+^) in lung tissues. (L) Cytokine levels of TNF‐α, IL‐6, and IL‐1β in BALF supernatants measured by ELISA. Data are presented as mean ± SD. Flow cytometry and ELISA: n = 5. Statistical analysis was performed using one‐way ANOVA with Tukey's post‐hoc test; ns, not significant; *p < 0.05, **p < 0.01, ***p < 0.001.

To further clarify the therapeutic advantage of CR_8_L_10_@CAT and determine an appropriate working dose for pulmonary administration, we established a mouse model of acute lung injury (ALI) by intratracheal instillation of LPS [38] (Figure 6C). CR_8_L_10_@CAT was administered at 2.0 mg/mL, 1.0 mg/mL, and 0.5 mg/mL in the lung model. Increasing the dose of CR_8_L_10_@CAT progressively enhanced the reduction of pro‐inflammatory cytokines, with the 2.0 mg/mL group showing the strongest therapeutic response. In addition, to further evaluate the therapeutic advantage of CR_8_L_10_@CAT in the ALI model, we compared it with free CAT as a non‐mucoadhesive control, R_8_L_10_@CAT as a cationic control, and dexamethasone (DEX) as a positive drug control. As shown in Figure S22, both free CAT and R_8_L_10_@CAT only partially reduced the levels of pro‐inflammatory cytokines, including TNF‐α, IL‐1β, and IL‐6, whereas CR_8_L_10_@CAT achieved a much stronger suppression of these cytokines. Notably, compared with the positive‐control drug DEX, CR_8_L_10_@CAT reduced these inflammatory mediators more effectively. These results confirm the therapeutic advantage of CR_8_L_10_@CAT in the ALI model (Figure S22). Taken together, these results support 2 mg/mL as the working dose for subsequent pulmonary therapeutic studies.

To validate the therapeutic potential of CR_8_L_10_@CAT in pulmonary inflammation, at 4 h post‐LPS challenge, mice received a single intratracheal aerosolized administration of PBS, free CAT, or CR_8_L_10_@CAT at a CAT concentration of 2 mg/mL (Figure 6C). At 24 h post‐administration, we evaluated the therapeutic effects by lung wet‐to‐dry (W/D) ratio and bronchoalveolar lavage fluid (BALF) total protein, which are standard indicators of epithelial barrier disruption and pulmonary edema in ALI [40, 41]. As shown in Figure 6D, the lung W/D ratio and BALF total protein level were significantly increased in the model group, indicating successful establishment of the ALI model with alveolar‐capillary barrier damage and pulmonary edema. Compared with the PBS‐treated model group, the CAT group displayed a modest decrease in those values, while CR_8_L_10_@CAT inhalation effectively reduced the lung W/D ratio and BALF total protein levels, with values nearly approaching those of the healthy group (Figure 6D). These results suggest that the mucoadhesive nanoantioxidant effectively protected the alveolar‐capillary barrier and substantially mitigated pulmonary edema in ALI.

Next, ROS generation and apoptotic cells were evaluated by DHE and TUNEL staining, respectively. Similar to the therapeutic outcomes observed in the IC/BPS model, CR_8_L_10_@CAT‐treated ALI mice also showed substantially reduced oxidative stress and decreased apoptosis of pulmonary epithelial cells (Figure 6E, F). In Figure S23, the H&E images of the PBS‐treated model group revealed obvious signs of interstitial edema, thickened alveolar septa, and disrupted alveolar architecture. Following treatment with CR_8_L_10_@CAT, these pathological abnormalities were markedly attenuated.

Afterwards, immune cells in BALF and lung tissues were analyzed by flow cytometry. The proportion of neutrophils (Ly6G^+^CD11b^+^) in BALF decreased in the CR_8_L_10_@CAT group (Figure 6G). Concurrently, the proportion of interstitial macrophages (F4/80^+^CD11b^+^) in lung tissues was markedly reduced with CR_8_L_10_@CAT treatment (Figure 6H). Furthermore, CR_8_L_10_@CAT treatment shifted macrophage polarization to an anti‐inflammatory M2 phenotype (Figure 6I), which was also confirmed by CLSM analysis of lung sections (Figure 6J). In parallel, flow cytometry revealed that CR_8_L_10_@CAT administration significantly reduced adaptive immune T cell (CD45^+^CD3^+^) counts in lung tissues (Figure 6K). Accordingly, the concentrations of pro‐inflammatory cytokines, such as IL‐6, TNF‐α, and IL‐1β, were all significantly reduced in BALF by CR_8_L_10_@CAT administration (Figure 6L). Consistently, CR_8_L_10_@CAT treatment also decreased the phosphorylation levels of NF‐κB p65 in lung tissues (Figure S24). The above findings demonstrate that CR_8_L_10_@CAT alleviates acute lung injury by suppressing immune cell recruitment and activation, as well as reducing pro‐inflammatory cytokine production. These results further suggest that this mucoadhesive nanoantioxidant strategy could be broadly applicable to manage different mucosal inflammatory diseases, holding great promise for clinical translation.

To further evaluate the systemic safety of CR_8_L_10_@CAT after pulmonary administration, serum biochemistry parameters and hematological indices were examined in mice treated with CR_8_L_10_@CAT at 2 mg/mL (therapeutic dose) or 4 mg/mL (twofold therapeutic dose). Consistent with observations in the bladder study, liver‐ and kidney‐related markers (ALT, AST, ALP, BUN) and hematological indices (WBC, RBC, HGB, HCT, MCV, MCH, MCHC, PLT) showed no obvious differences compared with healthy controls, indicating no detectable systemic toxicity or hematological disturbance and supporting the favorable safety profile of CR_8_L_10_@CAT (Figure S25).

## 3 Conclusions

In this work, we developed a mucoadhesive peptide‐enzyme nanoantioxidant, CR_8_L_10_@CAT, which was formed by self‐assembling the endogenous antioxidant enzyme catalase (CAT) with a cysteine‐modified amphiphilic peptide (CR_8_L_10_), for the treatment of mucosal inflammatory diseases. In this system, the cysteine residues in CR_8_L_10_ would mediate dynamic thiol‐disulfide exchange with mucin networks, resulting in strong adhesion of CR_8_L_10_@CAT to mucosal surfaces and prolonged residence under physiological clearance conditions. This prolonged mucosal retention enhances the effective local exposure of CAT at inflamed sites and supports sustained, high‐efficiency elimination of reactive oxygen species (ROS). Subsequently, we evaluated the therapeutic efficacy of CR_8_L_10_@CAT in two representative mucosal inflammation mouse models. In the IC/BPS model, intravesical CR_8_L_10_@CAT treatment achieved urine‐resistant bladder retention and efficiently scavenged excessive ROS in the bladder wall, thereby attenuating inflammatory cytokine production, restoring urothelial barrier integrity, and improving pain‐related voiding behavior. Notably, CR_8_L_10_@CAT produced more potent and durable therapeutic benefits than clinically used intravesical agents such as DMSO, HA, and HA/CS. Moreover, we demonstrated that the inhaled CR_8_L_10_@CAT with enhanced pulmonary retention could be used to treat ALI by scavenging ROS in pulmonary tissues and modulating the inflammatory microenvironment, confirming that this nanoantioxidant design can provide effective protection across anatomically distinct mucosal organs.

For mucosal inflammatory diseases, current clinical therapeutics such as instilled agents (e.g., DMSO, HA/CS) for IC/BPS treatment only provide partial and often transient control of disease activity. On one hand, physiological clearance mechanisms such as urinary flushing and mucociliary clearance would lead to short residence time of administered drugs on mucosal surfaces, preventing sustained local drug exposure. On the other hand, these therapies largely target downstream steps of the inflammatory cascade or passively support mucosal barrier repair and are therefore limited in their abilities to fundamentally alter the disease progression. Against this background, CR_8_L_10_@CAT is designed to overcome these shortcomings. First, the cysteine‐modified peptide carrier CR_8_L_10_ can undergo dynamic thiol–disulfide exchange with mucin networks, enabling active anchoring to the mucus layer, markedly prolonging local retention of the therapeutic agent. Second, CR_8_L_10_@CAT directly targets the common pathogenic feature of excessive ROS, an upstream disease driver in mucosal inflammatory diseases, so as to disrupt the ROS‐inflammation positive feedback loop. Compared with small‐molecule antioxidants that are rapidly consumed and cleared, catalase can provide a more sustained ROS‐scavenging effect with great biocompatibility. The excellent therapeutic efficacies observed in both bladder and lung mucosal inflammation models suggest that CR_8_L_10_@CAT may be a promising type of nano‐therapeutics to treat various mucosal inflammatory diseases.

## Conflicts of Interest

The authors declare no conflicts of interest.

## Supporting information




**Supporting File**: advs76390‐sup‐0001‐SuppMat.docx.

## Data Availability

The data that support the findings of this study are available from the corresponding author upon reasonable request.
