# Supplementary material for: Mucoadhesive Peptide‐Catalase Self‐Assembled Nano‐Formulation for Effective Treatment of Mucosal Inflammatory Diseases
Source: Adv Sci (Weinh). 2026 Jul 2;13(54):e76390. doi: 10.1002/advs.76390 (PMC13337007; doi:10.1002/advs.76390)
Supplement: Supplementary file 1 [file ADVS-13-e76390-s001.docx]

**Supplementary Information**

**Materials**

Lipopolysaccharide (LPS), protamine sulfate (PS), dithiothreitol (DTT), mucin (from porcine stomach), Cy5.5 NHS ester, ninhydrin, collagenase IV, DNase I, and ammonium molybdate were purchased from Sigma-Aldrich (St. Louis, MO, USA). Dimethyl sulfoxide (DMSO), sodium hyaluronate (HA), chondroitin sulfate (CS), N-ethylmaleimide (NEM), and citrate-stabilized gold nanoparticles (diameter ≈ 5 nm) were purchased from Sigma-Aldrich (St. Louis, MO, USA). Phosphate-buffered saline (PBS), Dulbecco’s Modified Eagle’s Medium (DMEM), penicillin/streptomycin, and fetal bovine serum (FBS) were obtained from Gibco BRL/Life Technologies (Grand Island, NY, USA). Catalase (CAT) was supplied by Hzymes Biotechnology Co., Ltd. (Wuhan, China). The peptides R_8_L_10_ and CR_8_L_10_ were synthesized by GL Biochem (Shanghai, China). Hoechst 33342, BCA assay kit, TUNEL assay kit, 2′,7′-dichlorofluorescein diacetate (DCFH-DA), 4′,6-diamidino-2-phenylindole (DAPI), and protease inhibitor cocktail were supplied by Beyotime (Shanghai, China). The catalase activity assay kit was acquired from Solarbio (Beijing, China). Mouse ELISA kits for TNF-α, IL-6, and IL-1β were sourced from Invitrogen (Carlsbad, CA, USA). Flow cytometry antibodies against CD45, CD11b, Ly6G, F4/80, CD86, CD206, CD3, CD11c, c-Kit, and FcεRIα were purchased from BioLegend (San Diego, CA, USA). Dihydroethidium (DHE) was obtained from MedChemExpress (Monmouth Junction, NJ, USA). OCT compound was procured from Servicebio (Wuhan, China). Primary antibodies for western blotting, including phosphorylated p65 (p-p65), total p65, and GAPDH, were purchased from Cell Signaling Technology (Danvers, MA, USA). qPCR reagents, including the reverse transcription kit and SYBR Green Master Mix, were obtained from Vazyme Biotech Co., Ltd. (Nanjing, China).

**Animals**

All procedures were approved by the Institutional Animal Care and Use Committee (IACUC) of Soochow University. C57BL/6 female mice (8-10 weeks) were used for bladder studies (retention, IC/BPS, behavior/voiding), and C57BL/6 male mice (8-10 weeks) were used for lung studies (ALI). Mice were purchased from GemPharmatech Co., Ltd. (Suzhou, China). Male New Zealand rabbits were obtained from Cavens Laboratory Animal Co., Ltd. (Changzhou, China). Animals were housed under SPF conditions with a standard light/dark cycle and ad libitum access to food and water, and acclimated for ≥1 week prior to experimentation. The animal experiments were conducted in accordance with ethical guidelines and received approval from the Institutional Animal Care and Use Committee at Soochow University. Approval number: SYXK(Su)2021-0073.

**Cell culture**

The murine macrophage cell line RAW264.7, murine lung epithelial cell line MLE-12, mouse bladder epithelial cells MB49, and human bronchial epithelial cell line BEAS-2B were acquired from the Cell Bank of the Chinese Academy of Sciences (Shanghai, China). All cell lines were cultured in high-glucose Dulbecco's Modified Eagle Medium (DMEM), supplemented with 10% fetal bovine serum (FBS) and 1% penicillin/streptomycin, and were incubated at 37°C in a humidified atmosphere containing 5% CO₂.

**Preparation of nanocomplexes**

The catalytic protein was prepared by dissolving CAT in PBS (pH 7.4) and dialyzing it against fresh PBS using a membrane (MWCO: 30 kDa) at 4°C for 4 hours. All PBS solutions were thoroughly deoxygenated by nitrogen bubbling, and dialysis was performed in tightly capped tubes under a nitrogen atmosphere to minimize oxygen exposure. The resulting solution was purified by passage through a 0.22 µm filter, and the CAT concentration was quantified using a BCA assay. Separately, the R_8_L_10_ and CR_8_L_10_ peptides were dissolved in deoxygenated ultrapure water. For nanocomplex formation, aqueous solutions of the peptides were combined with the purified CAT solution at designated weight ratios (e.g., peptide to CAT at 1:1, 5:1, 10:1, and 15:1). The mixtures were gently mixed and then allowed to stand at room temperature for 30 min to facilitate self-assembly, which were designated as R_8_L_10_@CAT and CR_8_L_10_@CAT. In addition, the surface thiol content of CR8L10@CAT nanocomplexes was determined using a commercial thiol assay kit.

**Characterization of nanocomplexes**

A comprehensive characterization of the nanocomplexes was performed using the following techniques. The hydrodynamic diameter, polydispersity index (PDI), and zeta potential were measured using a Zetasizer Nano ZS instrument. The morphology of the nanoparticles was examined by transmission electron microscopy (TEM) using a TF20 instrument. For fluorescence-based tracking, CAT was labeled by reacting with a 4-fold molar excess of Cy5.5 NHS ester in PBS buffer overnight at 4°C. The resulting conjugate (CAT-Cy5.5) was purified using a desalting column to remove unreacted dye, and its concentration was determined by BCA assay prior to the preparation of fluorescently labeled nanocomplexes. Free CAT and CR_8_L_10_@CAT were diluted in deionized water to a final CAT concentration of 0.1 mg/mL, and their zeta potentials were measured at 25°C. Each sample was analyzed in triplicate.

**Encapsulation efficiency determination *in vitro***

CR_8_L_10_@CAT were analyzed by ultrafiltration to quantify free CAT and calculate encapsulation efficiency (EE). Briefly, 100 μL of each sample (free CAT or CR_8_L_10_@CAT) was loaded into a 2 mL centrifugal ultrafiltration device (MWCO = 300 kDa), and centrifuged at 4,000 × g for 10 min at 4°C. The ultrafiltrate was collected and its volume was recorded. Catalase activity in the ultrafiltrate was determined by the A240 H₂O₂ decomposition assay. Encapsulation efficiency was calculated as:

$$EE(\%)=\left( 1 - \frac{U_{1}}{U_{0}} \right)\times100$$

where **U₁** is the total CAT activity recovered in the ultrafiltrate and **U₀** is the total CAT activity in the input sample prior to ultrafiltration.

**Stability *in vitro***

To evaluate colloidal and functional stability under bladder-relevant and protein-containing conditions, CR_8_L_10_@CAT was incubated in artificial urine or 10% FBS at 37°C. At predetermined time points (0, 12, 24, and 48 h), aliquots were collected for hydrodynamic size measurement by DLS and residual catalase activity analysis using the A240 assay after appropriate dilution into phosphate buffer. For lyophilization, freshly prepared CR_8_L_10_@CAT suspensions (equivalent CAT concentration 1 mg/mL) were aliquoted into glass vials, frozen at −80°C overnight, and lyophilized under vacuum for 12 h. The resulting dry powders were stored at 4°C. For reconstitution, lyophilized CR_8_L_10_@CAT powders were re-dispersed in PBS (pH 7.4) to the original volume by gentle shaking at room temperature for 15 min to obtain reconstituted samples (RS), which were then compared with freshly prepared samples (FS) in terms of hydrodynamic size, PDI, and CAT activity.

**AuNP binding assay and size measurement**

To assess the presence and accessibility of thiol groups on CR_8_L_10_@CAT, citrate-stabilized gold nanoparticles (AuNPs) were incubated separately with R_8_L_10_@CAT and CR_8_L_10_@CAT. Briefly, AuNP suspensions were mixed with R_8_L_10_@CAT or CR_8_L_10_@CAT and gently shaken at room temperature for 20 min. Bound AuNPs were removed by centrifugation, and the resulting samples were imaged by HAADF-STEM to visualize AuNP binding. In parallel, the hydrodynamic diameters of R_8_L_10_@CAT and CR_8_L_10_@CAT were measured by DLS before and after AuNP incubation to evaluate AuNP-induced size changes.

**Mucin adhesion assay in *vitro***

Mucin-coated ELISA plates were prepared by coating wells with mucin (10 mg/mL in PBS, 100 µL per well) overnight at 4°C. Plates were washed five times with PBST (PBS containing 0.1% Tween-20) and then incubated with 100 µL of CAT, R₈L₁₀@CAT, or CR₈L₁₀@CAT (final CAT concentration: 1 mg/mL) for 1 h at 37°C. After incubation, wells were washed five times with PBST to remove unbound materials. To evaluate the dependence of mucin binding on surface-accessible thiols, CR₈L₁₀@CAT was pretreated with N-ethylmaleimide (NEM) at different molar equivalents relative to surface thiols for 30 min at room temperature to partially block free thiols, followed by removal of excess NEM. Residual surface thiols were quantified using Ellman’s reagent (DTNB) according to the manufacturer’s protocol. The NEM-treated nanocomplexes were then applied to mucin-coated plates as described above, and mucin-retained catalase activity was quantified by the residual H₂O₂ assay (see below).

To specifically disrupt thiol–disulfide exchange and/or disulfide anchoring, a parallel set of wells was treated with 100 µL DTT (20 mM) for 30 min after nanocomplex incubation; control wells received PBS. Wells were then washed five times with PBST.

Hydrogen peroxide solution (1 M, 100 µL) was added to each well and allowed to react for 3 min at room temperature. The residual H₂O₂ concentration was determined using the ammonium molybdate colorimetric method, and the amount of catalase retained on the mucin layer was calculated accordingly.

**Evaluation of intracellular ROS in *vitro***

RAW264.7 cells were seeded in 12-well plates and then stimulated with H_2_O_2_ (200 μM) or LPS (100 ng/mL) and co-treated with PBS, CAT, or CR_8_L_10_@CAT for 12 hours. Subsequently, cells were incubated with 10 µM DCFH-DA at 37°C for 30 minutes. ROS levels were assessed qualitatively by confocal laser scanning microscopy (CLSM) and quantitatively by flow cytometry.

**Apoptosis assay in *vitro***

MLE-12, MB49, and BEAS-2B cells were seeded in 12-well plates and stimulated with LPS (100 ng/mL) for 12 hours along with PBS, CAT, or CR_8_L_10_@CAT. Unstimulated cells served as controls. After the incubation period, cells were collected, washed with PBS, and stained with Annexin V-FITC and propidium iodide (PI) for 20 minutes, protected from light. Apoptosis was analyzed by flow cytometry.

**Macrophage polarization and cytokine secretion assay in *vitro***

To evaluate the anti-inflammatory effects of the nanocomplexes, RAW264.7 cells were stimulated with LPS (100 ng/mL) or H_2_O_2_ (200 μM) and co-treated with PBS, free CAT, or CR_8_L_10_@CAT for 12 hours. After the incubation, the cell culture supernatants and cells were processed in parallel. The supernatants were collected by centrifugation, and the levels of secreted pro-inflammatory cytokines (TNF-α, IL-6, and IL-1β) were quantified using ELISA kits. Simultaneously, the harvested cells were stained with fluorescently labeled antibodies, including FITC-anti-F4/80, PE-anti-CD86, and APC-anti-CD206, and analyzed by flow cytometry to assess macrophage polarization. The M1 phenotype was identified as F4/80⁺CD86⁺ cells, and the M2 phenotype was defined as F4/80⁺CD206⁺ cells.

**Bladder retention study *in vivo***

Female C57BL/6 mice were anesthetized, and 50 µL of CAT-Cy5.5, R_8_L_10_@CAT-Cy5.5, or CR_8_L_10_@CAT-Cy5.5 (CAT concentration: 1 mg/mL) was intravesically instilled via catheterization. The solution was retained for 2 hours, after which mice were allowed to void freely. At 24 hours post-instillation, bladders were harvested for IVIS imaging. For mechanistic studies, mouse bladders were exposed intravesically to DTT (10 mM) for 30 minutes before harvest. Bladder cryosections were prepared and imaged by CLSM.

To characterize bladder exposure, systemic leakage, and organ biodistribution after intravesical administration, female C57BL/6 mice were intravesically instilled with free CAT or CR_8_L_10_@CAT at an equal CAT-equivalent dose (1 mg/mL, 50 μL). The formulations were retained in the bladder for 2 h, after which the catheter was removed and mice were allowed to void freely; this time point was defined as 0 h. At 2, 6, 12, 24, and 48 h thereafter, bladder tissues and serum were collected. For biodistribution analysis, at 24 h after administration, major organs including heart, liver, spleen, lung, kidney, and bladder were harvested, weighed, and homogenized. Catalase activity in tissue homogenates and serum samples was first measured using a commercial catalase activity assay kit according to the manufacturer’s instructions, and the CAT concentration was then calculated based on a standard curve generated with known CAT concentrations.

***Ex vivo* rabbit bladder adhesion study**

Freshly excised rabbit bladders were incubated with R_8_L_10_@CAT-Cy5.5 or CR_8_L_10_@CAT-Cy5.5 for 2 hours at 37°C. After incubation with nanocomplexes, bladder tissues were treated with DTT (10 mM) for 30 min to disrupt disulfide-mediated adhesion, while the control group received PBS instead. After thorough washing with artificial urine, the bladder tissues were sectioned and visualized by confocal laser scanning microscopy (CLSM).

**Dose-response and control-comparison study in the IC/BPS model**

Female C57BL/6 mice were anesthetized and subjected to sequential intravesical instillation of PS followed by LPS on days 1, 5, 9, and 13 to establish the IC/BPS model. Briefly, PS (30 mg/mL, 50 μL per mouse) was first instilled into the bladder and retained for a defined period to disrupt the urothelial barrier and increase bladder mucosal permeability. After bladder emptying, LPS (4 mg/mL, 50 μL per mouse) was subsequently instilled to induce local inflammatory responses. To compare control formulations and determine an appropriate therapeutic dose, additional IC/BPS cohorts were randomly assigned to the following groups: Healthy, Untreated, CAT (1 mg/mL), R_8_L_10_@CAT (1 mg/mL), CR_8_L_10_@CAT (0.25 mg/mL), CR_8_L_10_@CAT (0.5 mg/mL), and CR_8_L_10_@CAT (1 mg/mL). All formulations were intravesically administered at 50 μL per mouse on days 15, 17, and 19. At the experimental endpoint, bladder tissues were harvested 48 h after the last instillation (day 21), homogenized, and analyzed for TNF-α, IL-1β, and IL-6 levels by ELISA.

Using the same IC/BPS model described above, mice were treated with intravesical instillations of PBS, free CAT, or CR_8_L_10_@CAT (equivalent CAT dose of 1 mg/mL, 50 μL per mouse) on days 15, 17, and 19. Therapeutic outcomes were assessed 48 h after the last instillation (day 21).

**Pain behavior and voiding function assessment *in vivo***

Mechanical allodynia of the pelvic region was assessed using the von Frey filament test. Mice were placed individually in transparent plexiglass chambers on an elevated wire mesh grid and allowed to acclimate for at least 30 minutes. A series of calibrated von Frey filaments (ranging from 0.04 g to 0.16 g) was applied perpendicularly to the suprapubic area with sufficient force to cause slight bending, held for 5 seconds. A positive response was defined as immediate licking or scratching of the stimulated area, sharp retraction of the abdomen, or jumping. The stimulus-response relationship was evaluated by plotting the response frequency against the corresponding filament force.

Spontaneous pain-related behaviors were evaluated in the absence of external stimulation. After a 30-min acclimation period, mice were observed for 15 min in individual chambers, and the cumulative number of predefined pain-related responses, including licking or grooming of the lower abdomen or urethral area and abdominal guarding, was recorded by an observer blinded to the treatment groups.

Voiding function was assessed by quantifying the void spot frequency. Individual mice were placed in laboratory cages lined with pre-weighed white filter paper for a duration of 2 hours, following a 30-minute acclimation period. During the test, mice were deprived of food and water. Subsequently, the filter paper was carefully removed and evenly sprayed with a 0.2% (w/v) ninhydrin solution in ethanol. The paper was then heated at 65°C for 5-10 minutes to develop the characteristic purple color at the sites of urine deposition. The number of distinct purple spots was counted for each mouse, and the total count over the 2-hour session was used as the measure of voiding frequency.

**Intravesical clinical comparator treatments in the IC/BPS model**

To compare CR_8_L_10_@CAT with clinically used intravesical agents, additional IC/BPS cohorts received DMSO, HA, or HA/CS as intravesical treatments after model establishment. Mice in the DMSO group were instilled with DMSO solution at a defined volume fraction (50% v/v, 50 μL per mouse). The HA group received sodium hyaluronate solution prepared at a clinically relevant concentration (20 mg/mL, 50 μL). The HA/CS group received a mixed solution containing sodium hyaluronate and chondroitin sulfate (16 and 20 mg/mL, respectively; 50 μL), formulated to approximate the composition of clinical GAG-replenishment products. For the extended treatment protocol shown in the schematic, mice in all treatment groups (PBS, DMSO, HA, HA/CS, and CR_8_L_10_@CAT) received intravesical instillations on days 15, 17, 19, 22, 24, and 26 (six instillations in total). In all groups, the instilled solution was retained in the bladder for 2 h via catheterization and then allowed to drain. Behavioral and voiding assessments were performed 24 h after the third instillation (day 22, defined as the 1-week time point) and 24 h after the sixth instillation (day 28, defined as the 2-week time point). Bladder wet weight and histological endpoints were evaluated at the end of the 2-week treatment period.

**Tissue harvest and processing *in vivo***

Bladders were harvested and weighed immediately after euthanasia. For histological analysis, tissues were processed by two independent methods. One portion was embedded in OCT compound and snap-frozen for cryosectioning. The remaining portion was fixed in 4% paraformaldehyde, followed by dehydration and paraffin embedding.

**Immunofluorescence staining *in vivo***

Cryosections were used for fluorescence-based assessments. To detect reactive oxygen species (ROS), sections were incubated with 10 μM dihydroethidium (DHE) at 37°C for 30 minutes in the dark. Apoptosis was evaluated using a TUNEL assay kit according to the manufacturer’s instructions. All sections were counterstained with DAPI (1 μg/mL) for nuclear visualization. After mounting with anti-fade medium, images were acquired using confocal laser scanning microscopy (CLSM) under consistent parameters.

**Histochemical staining *in vivo***

Paraffin-embedded tissues were sectioned at 4 μm thickness. For general histopathology, sections were stained with hematoxylin and eosin (H&E) to assess tissue architecture, edema, and inflammatory infiltration. Mast cells were identified by toluidine blue staining of adjacent sections. All slides were examined under a light microscope.

**Systemic safety evaluation after intravesical administration**

To evaluate systemic safety after intravesical administration, healthy female C57BL/6 mice were treated with CR_8_L_10_@CAT at the therapeutic dose (1 mg/mL) or a twofold higher dose (2 mg/mL) under the same dosing schedule used in the efficacy study. At the experimental endpoint, blood samples were collected for serum biochemical and hematological analyses. Serum levels of alanine aminotransferase (ALT), aspartate aminotransferase (AST), alkaline phosphatase (ALP), and blood urea nitrogen (BUN) were measured using commercial assay kits. Whole-blood hematological parameters, including white blood cells (WBC), red blood cells (RBC), hemoglobin (HGB), hematocrit (HCT), mean corpuscular volume (MCV), mean corpuscular hemoglobin (MCH), mean corpuscular hemoglobin concentration (MCHC), and platelets (PLT), were analyzed according to standard procedures.

**qPCR validation of RNA-seq findings**

To validate bladder epithelial barrier–related genes identified by RNA-seq, total RNA was extracted from bladder tissues using a commercial RNA isolation kit according to the manufacturer’s instructions, and cDNA was synthesized using a reverse transcription kit. Quantitative real-time PCR (qPCR) was performed using SYBR Green Master Mix on a real-time PCR system. The relative mRNA expression levels of Upk1a and Upk3a were normalized to GAPDH and calculated using the 2^−ΔΔCt^ method.

**Western blot validation of NF-κB signaling in bladder tissues**

To validate NF-κB signaling in bladder tissues, bladder samples were homogenized in RIPA lysis buffer containing protease and phosphatase inhibitors. Total protein concentrations were determined using a BCA protein assay kit. Equal amounts of protein were separated by SDS–PAGE and transferred onto PVDF membranes. After blocking, the membranes were incubated overnight at 4°C with primary antibodies against phosphorylated p65 (p-p65), total p65, and GAPDH, followed by incubation with the corresponding HRP-conjugated secondary antibodies. Protein bands were visualized using an enhanced chemiluminescence detection system.

**Flow cytometric analysis *in vivo***

For immune profiling, bladder tissues were minced and digested in a solution of collagenase IV (1 mg/mL) and DNase I (100 μg/mL) at 37°C for 45–60 minutes to generate single-cell suspensions. After red blood cell lysis and filtration through a 70 μm strainer, cells were stained with fluorescently conjugated antibodies for 30 minutes at 4°C. The following immune populations were identified by flow cytometry: mast cells (CD45⁺c-Kit⁺FcεRIα⁺), neutrophils (CD45⁺CD11b⁺Ly6G⁺), macrophages (CD45⁺CD11b⁺F4/80⁺), and T cells (CD45⁺CD3⁺). Macrophage subsets were further defined as M1-like (CD86⁺) and M2-like (CD206⁺).

**Cytokine measurement by ELISA *in vivo***

Bladder tissues were homogenized in cold PBS supplemented with protease inhibitors. The homogenates were centrifuged, and supernatants were collected. Levels of TNF-α, IL-6, and IL-1β were quantified using commercial ELISA kits according to the manufacturer’s protocols.

**RNA sequencing and bioinformatic analysis *in vivo***

Total RNA was isolated from the bladder tissues of PBS-treated IC/BPS model mice and CR_8_L_10_@CAT-treated IC/BPS mice using RNAiso Plus reagent. Bladders were harvested 48 h after the last intravesical treatment. The quality and integrity of the RNA were verified. Sequencing libraries were constructed and sequenced on an Illumina platform by Beijing Genomics Institute (BGI, China). Bioinformatics processing of the raw data included quality control, alignment to the reference genome, and quantification of gene expression levels. Differentially expressed genes (DEGs) between groups were identified based on a threshold of an absolute log2 fold change (|log2FC|) ≥ 1 and an adjusted p-value (FDR) < 0.05. Functional enrichment analysis of the DEGs was performed using the Gene Ontology (GO) database.

**Acute lung injury (ALI) therapeutic study**

Male C57BL/6 mice were anesthetized and administered LPS (5 mg/kg in 50 μL PBS) via intratracheal instillation to induce ALI. To compare control formulations and determine an appropriate working dose for pulmonary administration, additional ALI cohorts were assigned to the following groups: Healthy, Untreated, CAT (2 mg/mL), R_8_L_10_@CAT (2 mg/mL), CR_8_L_10_@CAT (0.5 mg/mL), CR_8_L_10_@CAT (1 mg/mL), CR_8_L_10_@CAT (2 mg/mL), and DEX (1 mg/mL). At 4 h after LPS challenge, mice received a single pulmonary administration of the indicated formulations under the same dosing regimen. At the experimental endpoint, lung tissues were harvested 24 h after treatment, homogenized, and analyzed for TNF-α, IL-1β, and IL-6 levels by ELISA.

Using the same ALI model described above, male C57BL/6 mice were treated with a single pulmonary administration of PBS, free CAT, or CR_8_L_10_@CAT at an equal CAT-equivalent dose of 2 mg/mL, 4 h after LPS challenge. Mice were euthanized 24 h after treatment for subsequent analyses.

**ALI model and treatment *in vivo***

Male C57BL/6 mice were anesthetized and administered LPS (5 mg/kg in 50 µL PBS) via intratracheal instillation to induce ALI. At 4 h post-LPS challenge, mice received a single intratracheal aerosolized administration of PBS, free CAT, or CR_8_L_10_@CAT at an equivalent CAT dose of 2 mg/mL. Mice were euthanized 24 hours after treatment for analysis.

**Lung exposure, systemic leakage, and biodistribution of active CAT after pulmonary administration**

CAT-Cy5.5 or CR_8_L_10_@CAT-Cy5.5 was administered via intratracheal instillation. Lungs were harvested 24 hours post-administration for IVIS imaging and CLSM analysis of cryosections. To characterize local lung exposure, systemic leakage, and organ biodistribution after pulmonary administration, male C57BL/6 mice received a single pulmonary administration of free CAT or CR_8_L_10_@CAT at an equal CAT-equivalent dose (2 mg/mL, 50 μL). At 2, 6, 12, 24, and 48 h after administration, lung tissues and serum were collected. For biodistribution analysis, at 24 h after administration, major organs including heart, liver, spleen, lung, and kidney were harvested, weighed, and homogenized. Catalase activity in tissue homogenates and serum samples was measured using a commercial catalase activity assay kit according to the manufacturer’s instructions. The measured activity values were then converted to CAT concentrations using a standard curve generated with CAT solutions of known concentrations.

**Evaluation of ALI therapeutic efficacy *in vivo***

Therapeutic efficacy was assessed by multiple endpoints. Bronchoalveolar lavage fluid (BALF) was collected and its total protein concentration was measured. The lung wet-to-dry weight ratio was determined to quantify edema. Lung tissues were processed for H&E staining, DHE staining (ROS), and TUNEL assay (apoptosis). For immune cell analysis, single-cell suspensions from BALF and lung tissues were stained with fluorescent antibodies and analyzed by flow cytometry. Immune populations were defined as follows: neutrophils (CD11b⁺Ly6G⁺), interstitial macrophages (CD11b⁺F4/80⁺), alveolar macrophages (CD11c⁺F4/80⁺), M1-like macrophages (F4/80⁺CD86⁺), M2-like macrophages (F4/80⁺CD206⁺), and T cells (CD45⁺CD3⁺). Cytokine levels (TNF-α, IL-6, IL-1β) in BALF supernatant were measured by ELISA. NF-κB signaling was assessed by Western blot using antibodies against phosphorylated p65 and total p65.

**Systemic safety evaluation after pulmonary administration**

To evaluate systemic safety after pulmonary administration, healthy male C57BL/6 mice were treated with CR_8_L_10_@CAT at the therapeutic dose or a twofold higher dose under the same dosing regimen used in the efficacy study. At the experimental endpoint, blood samples were collected for serum biochemical and hematological analyses. Serum levels of alanine aminotransferase (ALT), aspartate aminotransferase (AST), alkaline phosphatase (ALP), and blood urea nitrogen (BUN) were measured using commercial assay kits. Whole-blood hematological parameters, including white blood cells (WBC), red blood cells (RBC), hemoglobin (HGB), hematocrit (HCT), mean corpuscular volume (MCV), mean corpuscular hemoglobin (MCH), mean corpuscular hemoglobin concentration (MCHC), and platelets (PLT), were analyzed according to standard procedures.

**Statistical analysis**

Statistical analysis was conducted using Prism 10 software (GraphPad). All data are expressed as the mean ± standard deviation (SD). Comparisons between multiple groups were performed using one-way analysis of variance (ANOVA) followed by Tukey's post hoc test. Statistical significance was defined as *p < 0.05, **p < 0.01, and ***p < 0.001.

**Supporting Information**

**Fig. S1*.*** Zeta potential (mV) of free CAT and CR₈L₁₀@CAT.

**Fig. S2.** Dilution stability of CR_8_L_10_@CAT nanocomplexes over a broad CR_8_L_10_ concentration range.

**Fig. S3.** Time course of H₂O₂ consumption catalyzed by free CAT and CR_8_L_10_@CAT (10:1).

**Fig. S4.** Hydrodynamic diameter and catalase activity of CR_8_L_10_@CAT after incubation in artificial urine at 37°C for 0, 12, 24, and 48 h.

**Fig. S5.** Hydrodynamic diameter and catalase activity of CR_8_L_10_@CAT after incubation in 10% FBS at 37°C for 0, 12, 24, and 48 h.

**Fig. S6.** Hydrodynamic size distributions of CR_8_L_10_ nanocomplexes formed with SOD (32 kDa), BSA (66 kDa), and IgG (150 kDa).

**Fig. S7.** Thiol-labeling analysis of reducible thiol-mediated linkages between CR₈L₁₀@CAT and mucin.

**Fig. S8.** RAW264.7 cells were stimulated with H₂O₂ (200 μM) and co-incubated with PBS, free CAT, or CR₈L₁₀@CAT for 12 h. Groups: untreated (G1), H₂O₂ + PBS (G2), H₂O₂ + CAT (G3), H₂O₂ + CR₈L₁₀@CAT (G4). CLSM images and quantification of intracellular ROS in RAW264.7 cells stained with Hoechst (nuclei, blue) and DCFH-DA (ROS, green) (top). Flow-cytometric analysis of intracellular ROS in RAW264.7 cells using DCFH-DA (bottom). Data are presented as means ± SD (n = 3). Scale bar: 20 µm.

**F****ig. S9.** Apoptosis analysis in BEAS-2B (left) and MB49 (right) by Annexin V–FITC/PI staining. Groups: untreated (G1), LPS + PBS (G2), LPS + CAT (G3), LPS + CR_8_L_10_@CAT (G4); Data are presented as means ± SD (n = 3).

**Fig. S10.** The concentration of CAT in bladder tissue (ng/g) at 2, 6, 12, 24, and 48 h after intravesical instillation of free CAT or CR₈L₁₀@CAT (2-h dwell followed by bladder emptying, defined as 0 h). Data are presented as mean ± SD (n = 3).

**Fig. S11.** The concentration of CAT in major organs after intravesical administration of free CAT or CR₈L₁₀@CAT, including heart, liver, spleen, lung, kidney, and bladder. Data are presented as mean ± SD (n = 3).

**Fig. S12.** Comparison of control formulations and dose-dependent anti-inflammatory effects of CR₈L₁₀@CAT in the IC/BPS model. Data are presented as means ± SD (n = 3).

**Fig. S13.** Stimulus–response curves of positive responses to graded von Frey filament forces in healthy mice, untreated IC/BPS mice, and IC/BPS mice treated with dimethyl sulfoxide (DMSO), hyaluronic acid (HA), hyaluronic acid/chondroitin sulfate (HA/CS), or CR_8_L_10_@CAT after (top) 1 week and (bottom) 2 weeks of continuous intravesical instillation. Groups: (G1) healthy control mice, (G2) IC/BPS mice treated with PBS, (G3) IC/BPS mice treated with DMSO, (G4) IC/BPS mice treated with HA, (G5) IC/BPS mice treated with HA/CS, and (G6) IC/BPS mice treated with CR_8_L_10_@CAT; Data are presented as means ± SD (n = 5).

**Fig. S14.** Gross morphology of bladders from different treatment groups in the IC/BPS model. Representative images of excised bladders collected at the end of the 2-week treatment period from (G1) healthy control mice, (G2) IC/BPS mice treated with PBS, (G3) IC/BPS mice treated with DMSO, (G4) IC/BPS mice treated with HA, (G5) IC/BPS mice treated with HA/CS, and (G6) IC/BPS mice treated with CR_8_L_10_@CAT.

**Fig. S15. Systemic safety evaluation of CR₈L₁₀@CAT after intravesical administration.** (A) Serum biochemical parameters, including ALT, AST, ALP, and BUN. (B) Hematological indices, including WBC, RBC, HGB, HCT, MCV, MCH, MCHC, and PLT. Healthy mice were treated with CR₈L₁₀@CAT at the therapeutic dose or a twofold higher dose. Data are presented as means ± SD (n = 3).

**Fig. S16.** Principal component analysis (PCA) of bladder RNA-seq data from PBS-treated IC/BPS mice (G2) and CR_8_L_10_@CAT-treated IC/BPS mice (G4).

**Fig. S17.** qPCR validation of bladder epithelial barrier–related genes. Groups: Healthy (G1), LPS/PS + PBS (G2), LPS/PS + CAT (G3), LPS/PS + CR_8_L_10_@CAT (G4). Data are presented as mean ± SD (n = 3).

**Fig. S18.** Western blot validation of NF-κB signaling in bladder tissues. Groups: Healthy (G1), LPS/PS + PBS (G2), LPS/PS + CAT (G3), LPS/PS + CR_8_L_10_@CAT (G4).

**Fig. S19.** The concentration of CAT in lung tissue (ng/g) at 2, 6, 12, 24, and 48 h after inhalation of free CAT or CR₈L₁₀@CAT. Data are presented as mean ± SD (n = 3).

**Fig. S20.** The concentration of CAT in major organs after pulmonary administration of free CAT or CR₈L₁₀@CAT, including heart, liver, spleen, lung, and kidney. Data are presented as means ± SD (n = 3).

**Fig. S21.** Quantitative analysis of pulmonary fluorescence after intratracheal administration of CAT-Cy5.5 or CR_8_L_10_@CAT-Cy5.5. Data are presented as means ± SD (n = 3).

**Fig. S22.** Comparison of control formulations and dose-dependent anti-inflammatory effects of CR₈L₁₀@CAT in the ALI model. Data are presented as means ± SD (n = 3).

**Fig. S23.** H&E staining of lung tissue; Groups: Healthy (G1), LPS + PBS (G2), LPS + CAT (G3), LPS + CR_8_L_10_@CAT (G4) (Scale bar, 100 μm).

**Fig. S24.** Western blot analysis of NF-κB signaling in lung tissues. All samples were loaded with equal amounts of protein in the same experiment. Groups: Healthy (G1), LPS + PBS (G2), LPS + CAT (G3), and LPS + CR_8_L_10_@CAT (G4).

**Fig. S25. Systemic safety evaluation of CR₈L₁₀@CAT after pulmonary administration**. (A) Serum biochemical parameters, including ALT, AST, ALP, and BUN. (B) Hematological indices, including WBC, RBC, HGB, HCT, MCV, MCH, MCHC, and PLT. Healthy mice were treated with CR₈L₁₀@CAT at the therapeutic dose or a twofold higher dose. Data are presented as means ± SD (n = 3).
